# Control technologies to prevent aerosol-based disease transmission in animal agriculture production settings: a review of established and emerging approaches

**DOI:** 10.3389/fvets.2023.1291312

**Published:** 2023-11-14

**Authors:** Hui Ouyang, Lan Wang, Deepak Sapkota, My Yang, José Morán, Li Li, Bernard A. Olson, Mark Schwartz, Christopher J. Hogan, Montserrat Torremorell

**Affiliations:** ^1^Department of Mechanical Engineering, University of Minnesota, Minneapolis, MN, United States; ^2^Department of Mechanical Engineering, University of Texas-Dallas, Richardson, TX, United States; ^3^Department of Veterinary Population Medicine, University of Minnesota, Saint Paul, MN, United States; ^4^Schwartz Farms, Sleepy Eye, MN, United States

**Keywords:** bioaerosol control, airborne pathogen transmission, swine health, bioaerosol control technology, airborne infection control, bioaerosol, livestock biosecurity, agricultural biosecurity

## Abstract

Transmission of infectious agents via aerosols is an ever-present concern in animal agriculture production settings, as the aerosol route to disease transmission can lead to difficult-to-control and costly diseases, such as porcine respiratory and reproductive syndrome virus and influenza A virus. It is increasingly necessary to implement control technologies to mitigate aerosol-based disease transmission. Here, we review currently utilized and prospective future aerosol control technologies to collect and potentially inactivate pathogens in aerosols, with an emphasis on technologies that can be incorporated into mechanically driven (forced air) ventilation systems to prevent aerosol-based disease spread from facility to facility. Broadly, we find that control technologies can be grouped into three categories: (1) currently implemented technologies; (2) scaled technologies used in industrial and medical settings; and (3) emerging technologies. Category (1) solely consists of fibrous filter media, which have been demonstrated to reduce the spread of PRRSV between swine production facilities. We review the mechanisms by which filters function and are rated (minimum efficiency reporting values). Category (2) consists of electrostatic precipitators (ESPs), used industrially to collect aerosol particles in higher flow rate systems, and ultraviolet C (UV-C) systems, used in medical settings to inactivate pathogens. Finally, category (3) consists of a variety of technologies, including ionization-based systems, microwaves, and those generating reactive oxygen species, often with the goal of pathogen inactivation in aerosols. As such technologies are typically first tested through varied means at the laboratory scale, we additionally review control technology testing techniques at various stages of development, from laboratory studies to field demonstration, and in doing so, suggest uniform testing and report standards are needed. Testing standards should consider the cost–benefit of implementing the technologies applicable to the livestock species of interest. Finally, we examine economic models for implementing aerosol control technologies, defining the collected infectious particles per unit energy demand.

## 1. Introduction

Airborne transmission of infectious agents has been documented or suspected for many years (1), and airborne pathogens cause some of the most devastating, costly, and difficult-to-control diseases. Examples of airborne pathogens in humans include *Mycobacterium tuberculosis*, SARS-CoV-2, respiratory syncytial virus (RSV), and influenza virus (2–4). Airborne transmitted pathogens in livestock include porcine reproductive respiratory syndrome virus (PRRSV), influenza A virus (IAV), high pathogenic avian influenza (HPAI), foot and mouth disease (FMD), *Mycoplasma hyopneumoniae*, classical swine fever (CSF), and porcine epidemic diarrhea (PED) viruses (5–8). Each of these animal pathogens has been responsible for causing devastating losses, in particular when causing disease in regions with high farm densities, due to the pathogens’ abilities to spread rapidly and, in some instances, cause zoonotic infections. The importance of having effective disease control strategies will continue as population growth expands (9), demand for animal protein increases (10), and feeding the world in a sustainable manner is central to food security. The cost of diseases to food animal production is significant and it cannot be ignored. For example, the 2014–2015 outbreak of HPAI was one of the most devastating foreign animal diseases in U.S. animal history, and containing it cost nearly $850 million dollars in obligated response activities and indemnity payments (11). In swine, PRRSV costs the North American swine industry more than $581 million dollars a year (12, 13).

**Figure 1:**
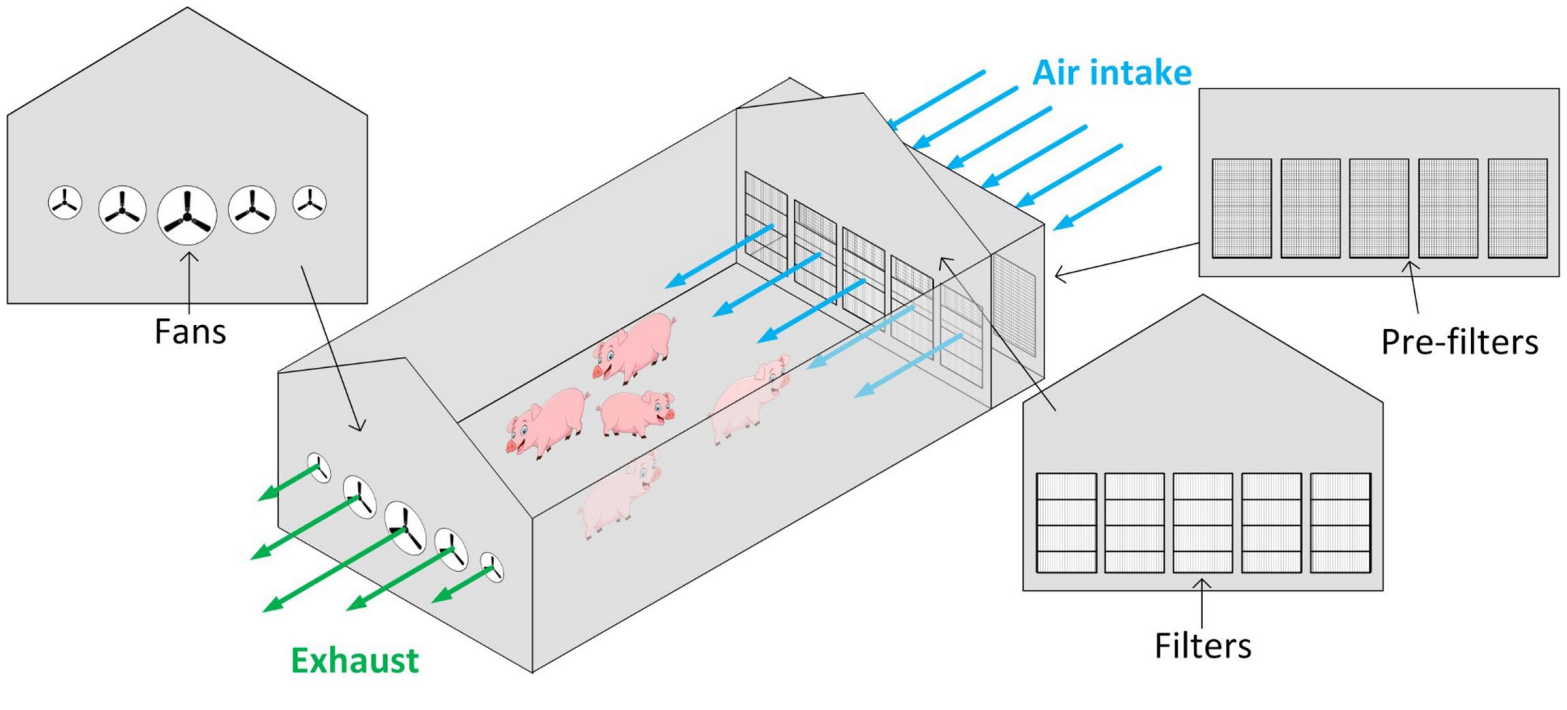
Example of a negative pressure swine barn ventilation system.

There is a need to have effective strategies to control the spread of bioaerosols, i.e., aerosols containing viable pathogens, in modern animal agriculture production settings. Animals are commonly raised in confined, naturally or mechanically ventilated buildings (see Figure 1), with high population densities. Over time, producers have developed measures to prevent disease introduction and disease spread to protect the health of animal populations. However, most of those measures have focused on minimizing disease risks through vaccination programs or biosecurity protocols that mitigate risks of contaminated fomites or infected animals (14, 15). Examples of such measures include employees showering in and out of farms, cleaning and disinfection of transport equipment, disinfection of materials prior to farm entry, and isolation and testing of replacement animals. These measures, although effective against many diseases, have not been sufficient to prevent the introduction (bioexclusion) and spread of airborne viruses (biocontainment).

There is also evidence of long-distance airborne transmission of viruses. In pigs, for example, PRRSV can be transported over extended distances by virus-laden aerosols (16–18). Evidence of long-distance airborne transport of PRRSV out to 4.7 km and 9.1 km has been reported. (19) In FMD, airborne transmission was the probable cause of several historic outbreaks where the FMD virus was suspected to travel over distances of 60 to 500 km over bodies of water (20) and land (21). Influenza A virus, which is a threat to both animal and public health because of its ability to change, reassort and cause pandemics (22), has also been detected in air samples from rooms of experimentally infected pigs (23), in air samples inside animal buildings and in the exhaust air from infected farms and at 1 mile from infected farms (24), highlighting the potential for aerosol transmission in pigs and between farms. Furthermore, the airborne spread of the HPAI virus was also implicated in the spread of avian influenza under certain conditions (25–27).

Given the evidence of long-distance airborne transmission of diseases in animals, in particular for PRRSV in pigs, filtration of incoming air to swine facilities has been proposed and implemented as a means to reduce the risk for PRRSV introduction (28–31). Filtration involves the passage of incoming air through fibrous filters, which mechanically (in most circumstances) collect aerosol particles, with the collection efficiency dependent upon both the sizes of the particles and the properties of the filter. The use of air filtration has been tested under experimental conditions and over the course of a 4-year study period involving a model of a swine production region. Under the study conditions, airborne transmission of PRRSV to susceptible populations housed in filtered facilities was prevented 100% of the time (19). As a result, the use of air filtration in breeding herds has become widespread in the Midwestern US (30, 32). Farms with air filtration had decreased incidence of PRRSV infections and improved wean pig quality (29, 33).

Although useful to prevent the introduction of airborne pathogens, air filtration implementation in large commercial animal settings is costly and has challenges. Costs associated with filtration include not only the purchase and replacement of the filters themselves, but also the increased energy requirements and increased energy consumption by the blower, to drive flow through filters. HEPA and high MERV (minimum efficiency reporting value) rated filters are widely considered the gold standard of air treatment technologies for pathogen control. However, their high capital cost, need for regular maintenance, and creation of large pressure drops in air systems pose a barrier to industrial application. For example, the capital cost of a HEPA filtration system has been estimated at $1,500–$2000 per boar/sow (34).

For this reason, the types of filters employed need to be judiciously selected, to ensure that they are not only effective in disease spread mitigation but also that costs are not overly cumbersome. Outside the animal production industry, e.g., in hospital environments as well as in industrial environments, there are alternative control technologies, such as electrostatic precipitators (ESPs) and ultraviolet type C (UV-C) light-based devices implemented in both removing aerosol particles from the air and inactivating pathogens in aerosols. Furthermore, over the last several decades and additionally intensified during the COVID-19 pandemic, there are newer control technologies in the developmental stage that may eventually be scalable toward farm-scale airborne disease transmission mitigation. Previous reviews have focused on general biosecurity measures (35) without much emphasis on aerosols. Some exceptions include identifying aerosol pollutants and the design of ventilation systems (35, 36) in poultry and swine farms. In addition, La et al. (37), reviewed different methods for aerosol research in virus transmission including PRRSV and African swine fever virus. However, a comprehensive assessment of existing and emerging virus aerosol control technologies is missing in the literature. With the already available wide variety of filtration options and the growing number of alternative technologies, we presently intend to review technologies directed at removing or inactivating airborne pathogens with a focus on those technologies that aim to prevent transmission among animals and from animals to people. We present the working principles of both existing and emerging technologies and discuss them in the context of using them in food animal production settings, particularly through incorporation with mechanically ventilated systems. We also discuss methods to test the effectiveness of the technologies to remove or inactivate viable pathogens and identify opportunities for further development. Finally, we discuss the installation and maintenance costs associated with various technologies. Overall, we aim to spark interest in developing and implementing technologies that can help protect animal populations and people and that contribute to long-term disease control, sustainable animal production systems and food security.

## 2. Control technologies overview

**Figure 2:**
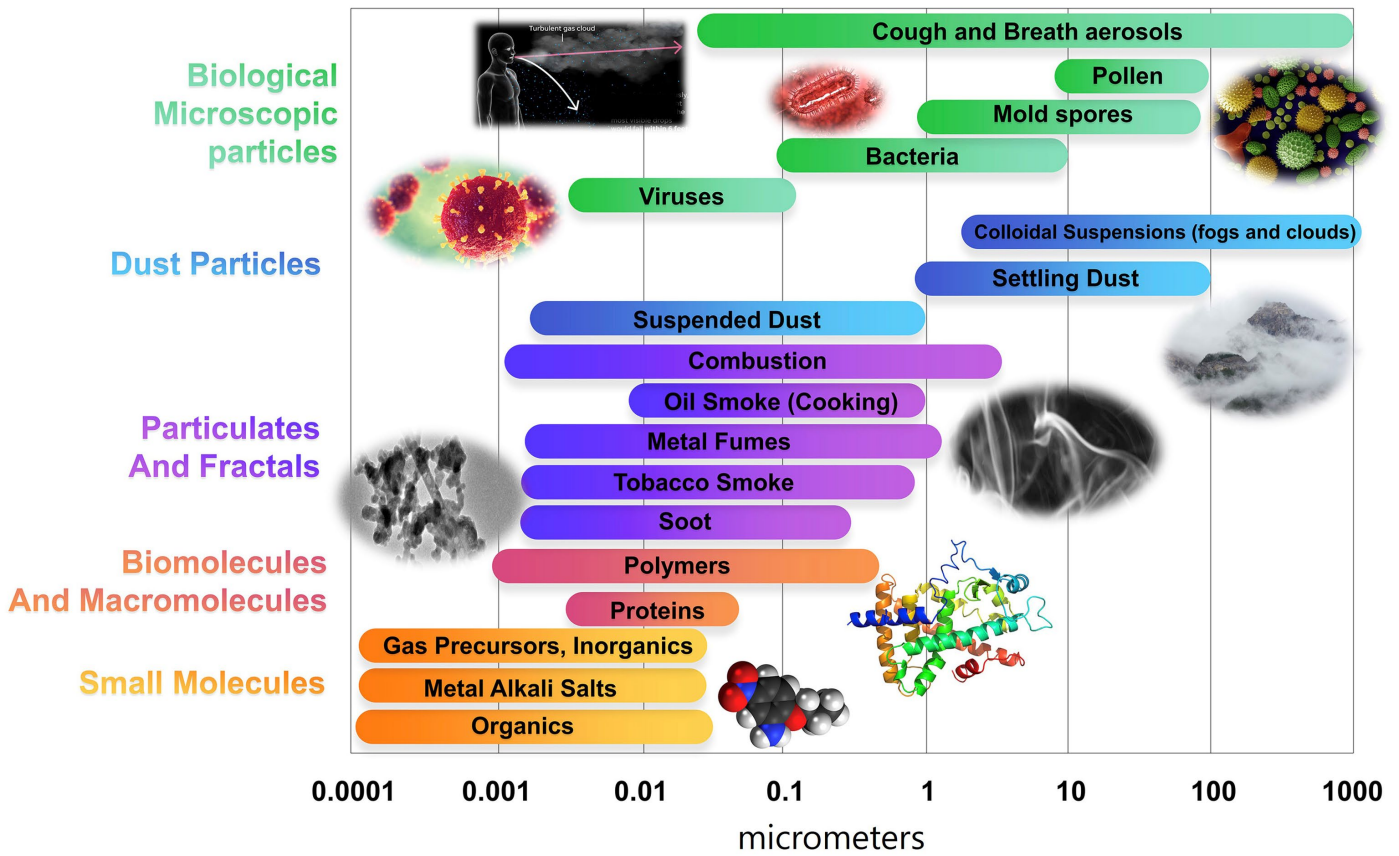
Characteristic aerosol particle size ranges, including bioaerosols and non-bioaerosols. Adapted from Larriba-Andaluz and Carbone (38).

A variety of technologies are available as engineering controls to remove and/or inactivate bioaerosols. The performance of these technologies depends on environmental and operational factors, including but not limited to the size and type of aerosol particles (which vary over orders of magnitude, as noted in Figure 2), operation period, temperature, and relative humidity. We focus specifically on discussing the capabilities of technologies applicable as engineering controls (i.e., applicable at the room scale or higher, and not as personal protective equipment or at the single-animal level) and largely technologies that can be incorporated into mechanically ventilated systems. We subdivide these technologies into three classes:

- Established and implemented technologies. Solely consists of fibrous filters, which are ubiquitously employed in reducing the spread of PRRSV, influenza, and a variety of other respiratory pathogens. We review the performance and ratings of filters as a baseline to compare to other technologies.
- Industrially- and medically-implementable technologies. Consisting of electrostatic precipitators and ultraviolet-C systems (both in-duct and upper room), they are commonly implemented in other industries but are less prevalent in livestock management. Nonetheless, because they have demonstrated scalable performance in aerosol particle removal and/or bioaerosol inactivation, they merit discussion and consideration toward agricultural biosecurity.
- Emerging technologies. Consisting of more recently developed technologies, typically at the laboratory or bench scale. Technologies in this category, which may include photocatalytic and plasma technologies, have not been tested at the scales (building sizes and flow rates) required for agricultural biosecurity and are hence several developmental steps away from direct implementation.

### 2.1. Established and implemented technologies – filters

**Figure 3:**
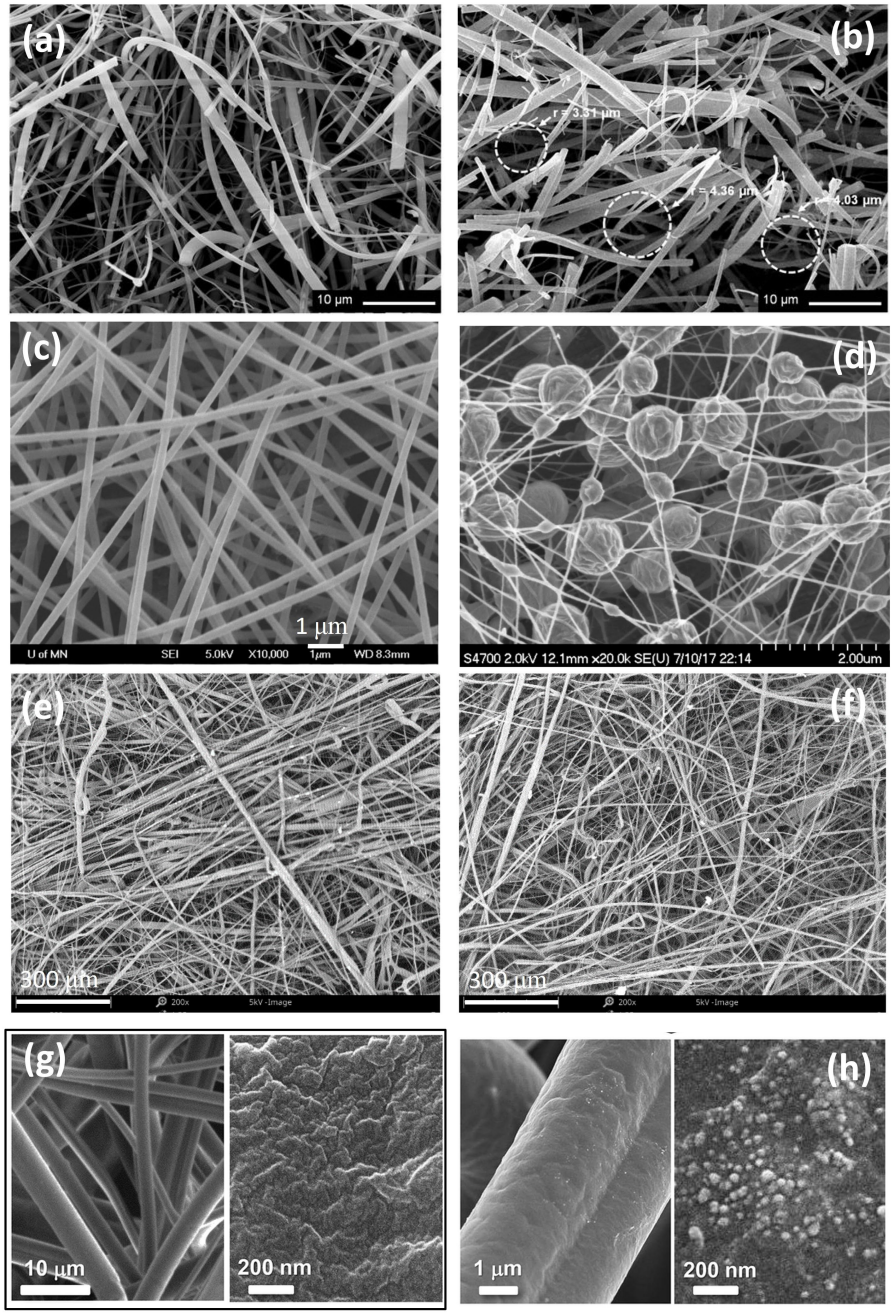
SEM images of typical quartz fiber filters with varying fiber diameters and varying “void” space dimensions [**(A,B)** adapted from Suárez-Peña et al. (39)]; a smooth nanofiber filter **(C)**; a beaded nanofiber filter **(D)** [**(C,D)** adapted from Kim et al. (40)], to be consistent with other images. TiO_2_ treated fiber filters [**(E,F)** adapted from Lou et al. (41)]; bare HEPA filter (left) with a zoom-in image of the fiber surface **(G)**, a tannic acid-treated HEPA filter (left) with a zoomed-in image of the fiber surface (right) **(H)** [**(G,H)** adapted from Kim et al. (42)].

**Figure 4:**
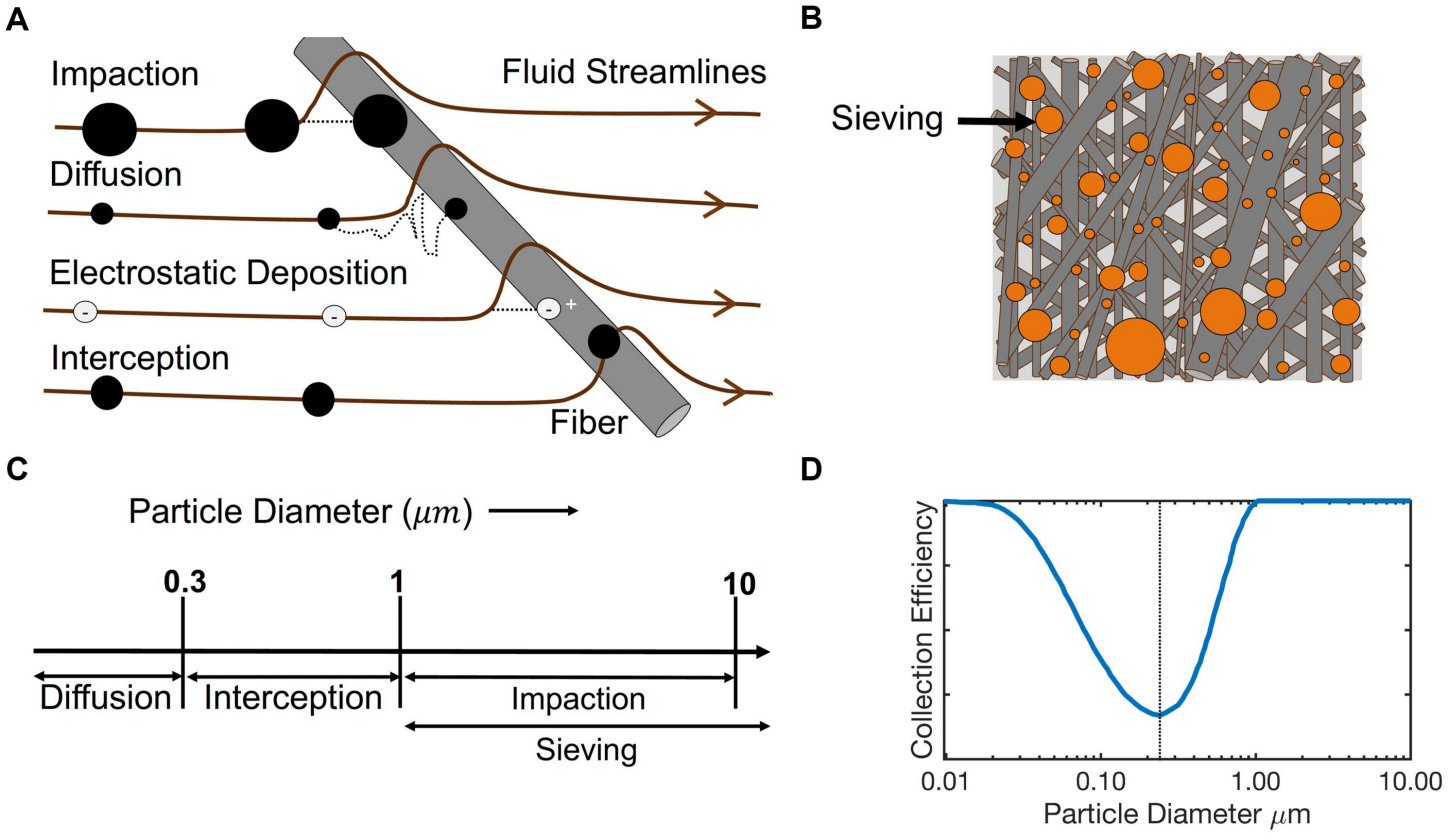
A depiction of different filtration mechanisms **(A,B)**; the approximate size ranges where mechanical filtration mechanisms act most prevalently **(C)**; and a characteristic collection efficiency curve shape for a fibrous filter, with the most penetrating size near 0.3 μm **(D)**.

If produced and installed properly, filters are very effective in removing a wide variety of particle types (dust, smoke, soot, bioaerosols) from flowing air streams, and are hence the most widely used technology in aerosol control. Filters are manufactured with multiple layers of non-woven fibers or with other materials, which are often cylindrical and randomly arranged inside the filter. As shown in Figure 3, the diameters of fibers often vary, with a filter typically described by the mean fiber diameter and a span of the fiber size distribution; given the random arrangement of fiber, the opening areas between cross-linked fibers vary in size as well within a filter (39, 43–45). The performance of filters varies greatly between filters and is described by the collection efficiency (η), which is defined as $\eta =1-C_{\text{down}}/C_{\text{up}}$, where *C*_down_ is the downstream particle concentration and *C*_up_ is the upstream particle concentration. When the filtration efficiency reaches 1 (100 %), all particles flowing through the filter are collected by it. The operational principle of filters is depicted in Figure 4A at the single filter fiber level. With a uniform incoming flow, the trajectory of air around the filter fiber is depicted by the streamlines. When the flow enters the filter, streamlines are curved and compressed near the surface of the fiber. Particles initially on streamlines approaching a filter fiber can be collected by several disparate mechanisms. First, as streamlines bend, particles with high mass-to-drag ratios (larger size and higher density), will not bend in trajectory with streamlines. Such particles inertially impact with fibers (impaction) (46). While impaction acts upon larger particles, particle diffusion coefficients increase with decreasing particle diameter (47), and high diffusivity particles have fluctuating motion, moving from streamline to streamline stochastically. Such diffusive motion can also lead to particle-fiber collisions and hence collection (diffusion) (48–50). Even particles that do not deviate from gas streamlines may collide with fibers through interception (51), which occurs for particles traveling along the streamline that approach a filter fiber at a distance less than the radius of the particle. As depicted in Figure 4C, impaction, diffusion, and interception are commonly the three mechanisms contributing the most to filter collection and enabling collection across orders of magnitude in particle diameter. However, as depicted in Figure 4D, because these three mechanisms are size-dependent, all filters have a collection efficiency curve with the most penetrating particle diameter (MPPD, the particle size of lowest collection efficiency) (52). The MPPD is typically in the 100 nm −400 nm diameter range for fibrous filters, as such particles have reduced diffusion coefficients but also insufficient inertia and size for collection by impaction or interception. The collection efficiency curve is affected by the filter face velocity (the speed of the flow), the diameter of the fibers and their size distribution (53), and other microstructural influences, such as solidity/porosity. A common assumption, which is reasonably valid in filters for aerosols, is that particles adhere irreversibly to fibers upon collision. Additional filtration mechanisms can also serve to collect particles in fibrous filters, including gravitational sedimentation for extremely large particles, sieving (Figure 4B), which occurs for particles too large to traverse through openings between fibers, and electrostatic effects, both for charged particles and uncharged particles (dielectrophoresis) within electret filters (54, 55), i.e., filters with intentionally introduced charge distributions on them.

To model collection by different mechanisms, it is common to invoke single fiber collection efficiency theories (56), which derive from more general collector theories for porous media and packed beds (57). However, while modeling filters can lead to predictions of efficiency that are qualitatively consistent with their performance; measurements are consistently required to properly define filter function. To evaluate the filter performance, ANSI and ASHRAE (American National Standards Institute and the American Society of Heating, Refrigerating and Air Conditioning Engineers) have established a standardized test that defines the Minimum Efficiency Reporting Values (MERV) rating for filters (the ANSI/ASHRAE 52.2–2017 standard). Table 1 shows how MERV ratings are assigned to filters based on their average filtration efficiencies in three different size ranges: 0.3 μm – 1 μm, 1 μm – 3 μm, and 3 μm – 10 μm. Note that smaller particles are not measured in the test, as doing so requires more specialized equipment than standard optical particle spectrometers, and the expectation is that below 0.3 μm, as particle diameters decrease, collection efficiency increases with a “U-shape” collection efficiency as shown in Figure 4D. As the MERV rating increases, the filtration efficiencies in all three ranges increase as well. For example, MERV 16 filters must reach 95% efficiency across all three ranges, while MERV 10 filters need only achieve 85% in the range of 3 μm – 10 μm, 50% in the range of 1 μm – 3 μm and have a negligible efficiency in the range of 0.3 μm – 1 μm. In general, using filters for bioaerosol removal, the higher the MERV rating, the better the removal efficiency. When a filter has filtration efficiencies in the three different size ranges which would lead to three different assigned MERV ratings, it is assigned to the lowest MERV, hence the MERV defines the “minimum efficiency” for the filter. Further, high-efficiency particulate air filters, known as HEPA filters, and Ultra-low particulate air (ULPA) filters meet requirements beyond the MERV rating scale. For example, HEPA filters must have a measured collection efficiency of 99.97% or greater for particles of 0.3 μm diameter and even higher collection efficiencies for particles smaller and larger in diameter.

**Table 1:** Collection efficiency requirements for minimum efficiency reporting value (MERV) rated filters.

ASHRAE standard 52.2 minimum efficiency reporting value (MERV) rating	Average particle collection efficiency in the size range of			Typical filter types
	Range 1 (0.3–1 μm)	Range 2 (1–3 μm)	Range 3 (3–10 μm)	
MERV 1–4	n/a	n/a	< 20%	Fiberglass and aluminum mesh
MERV 5	n/a	n/a	≥ 20%	Pre-filters and dust filters
MERV 6	n/a	n/a	≥ 35%	
MERV 7	n/a	n/a	≥ 50%	
MERV 8	n/a	≥ 20%	≥ 70%	
MERV 9	n/a	≥ 35%	≥ 75%	Home and commercial building filter
MERV 10	n/a	≥ 50%	≥ 80%	
MERV 11	≥ 20%	≥ 65%	≥ 85%	
MERV 12	≥ 35%	≥ 80%	≥ 90%	
MERV 13	≥ 50%	≥ 85%	≥ 90%	Hospital and clean room filter
MERV 14	≥ 75%	≥ 90%	≥ 95%	
MERV 15	≥ 85%	≥ 90%	≥ 95%	
MERV 16	≥ 95%	≥ 95%	≥ 95%	
MERV 17	99.97%	≥ 99%	≥ 99%	HEPA and ULPA
MERV 18	99.997%	≥ 99%	≥ 99%	
MERV 19	99.9997%	≥ 99%	≥ 99%	
MERV 20	99.99997%	≥ 99%	≥ 99%	

The MERV rating for a filter is determined by the ASHRAE/ANSI 52.2–2017 standard (58) and is based on “Range” corresponding to the filter’s lowest MERV value (59).

While at first glance it would appear higher MERV rated filters are always preferable, it is important to note that filters must be incorporated with forced air ventilation systems which are implemented to drive the pollutant air through the filter, and adding the filter into the ventilation system increases the pressure drop. The product of the pressure drop and air flow rate, divided by the electrical-to-mechanical energy efficiency of the blower used, gives the additional power (rate of energy use) associated with using filters. Therefore, a higher pressure drop requires a higher energy consumption to drive the same amount of flow. Filters with a higher MERV rating are typically made with smaller diameter fibers or made of higher solidity, often resulting in higher pressure drop (reduced permeability) (60), which increases energy cost or even requires replacement with a high-power blower/compressor to maintain the ventilation rate.

To quantify the filter’s overall performance using both the pressure drop (Δ*P*) and the collection efficiency (*CE*), the figure of merit (*FOM*) (43, 61) is introduced with the units of Pa^−1^ and calculated via the equation:


(1) $$FOM=\frac{\ln\left(\frac{1}{1-CE}\right)}{\Delta P}$$

The FOM, also commonly referred to as the quality factor, is a metric that enables a comparison of the energy costs associated with a filter use, in comparison to the collection it provides, and in general higher FOM filters are preferred in all applications; a lower pressure drop and high collection efficiency will result in a higher FOM. If two filters have the same collection/filtration efficiency, the filter with a higher FOM, thus a lower pressure drop, will be preferred. In practice, multiple banks are commonly installed along side walls, or multiple filter modules are installed in the attics in animal production facilities. In these installations, it is common to place two filters in series; the first a pre-filter of low MERV rating (to collect large dust particles and prevent the loading of the second filter) and a higher MERV-rated second filter, whose purpose is to collect micrometer-to-submicrometer particles. The FOM can also be computed for two filters used in series; in this case, the collection efficiency is the net efficiency considering both filters (upstream before the first filter, and downstream after the second filter), and the pressure drop is the sum of the pressure drops across the individual filters. Different products have also been marketed to improve FOM (improve collection efficiency and/or decrease the pressure drop). For example, electrostatic-enhanced (electret) filters can raise the figure of merit with a lower pressure drop and have been implemented in N95 respiratory face masks (62, 63). However, degradation of the electric charges over time will lower the filtration efficiency. Filters on the market are almost always pleated (54, 64) to increase the surface area of the filter, thus increasing the capacity for the collection of pollution. While pleating generally reduces the pressure drop, filters can be over pleated leading to an increase in pressure drop causing a lower FOM.

Unsurprisingly, as the collection efficiency is a function of particle diameter, the FOM is also size-dependent. In addition, in practice, the FOM varies with time, as the filter performance changes with loading [particles deposit onto and stay on the filter (65)]. While loading often leads to an increase in filtration efficiency, it typically leads to a proportionally greater increase in pressure drop (66). Thus, the replacement of filters periodically is required and the replacement frequency depends on the quality of the filter, flow condition, and the level of particle pollution in the air stream. It also depends on the filter arrangement; for example, in the noted case of a low MERV rated pre-filter and a higher MERV-rated main filter downstream, the pre-filter loads faster and needs more frequent replacement than the main filter. In general, for the same type of filter (same MERV rating and initial pressure drop), if the FOM of a filter decreases with time rapidly due to higher particle concentrations, the filter needs to be replaced more frequently.

For bioaerosol control and removal, even though the filtration efficiency is not strictly equivalent to bioaerosol removal efficiency (67), the filter still follows the same collection principles for bioaerosols as it does for other types of aerosols. For this reason, in collecting particles, the filter material is less significant than its structure and for aerosol filtration, it is typically not necessary to design filters out of materials promoting specific interactions with biomolecules. Bioaerosols which are typically larger than the size of bare viruses, fungi, and bacteria due to attachments of other materials, range from a few tens of nanometers to a few micrometers, Nevertheless, filters already target this range of particle diameters. Often, high MERV rating filters are needed to achieve high efficiency in bioaerosol removal from the air stream (68–70). At the same time, for bioaerosols, filters alone may not be sufficient: collected bacteria and fungi on filters may grow and eventually be resuspended into the air stream (71). In many instances for viruses, the half-lives on surfaces such as filter media have been measured to be hours to days (72, 73). The inactivation of collected bioaerosols upon capture by the filters is therefore also important to consider. To enhance the inactivation rate and prevent bacterial or fungal growth, one approach is to coat filters with different antimicrobial materials targeting collected bioaerosols. Numerous researchers have worked with a variety of coatings to this end, though with variable results on their effectiveness, and without a universal, commercialized approach presently available. Nanoparticle-coated filters with antimicrobial materials such as silver (Ag), copper (Cu), and carbon nanotubes. Among others have been recently examined. (74–77) Natural products including *Melaleuca alternifolia* (tea tree oil) coated filters have also been shown to inactivate filter-collected bioaerosols (78–81). For efficient bioaerosol control using surface-coated filters, the use of pre-filters and regular maintenance [to remove collected aerosols and avoid dust-loading (82)] and replacement would be required with a higher frequency, but this may prove prohibitively costly.

Despite the cost of high-quality filters and frequent replacements, filters are the most commonly used method to control bioaerosols, mainly because of their simple implementation, and proven effectiveness. Laboratory and field tests have been performed to evaluate the performance of filter-based air filtration systems in controlling bioaerosol transmission in livestock. Specifically, a group of researchers in Minnesota, USA conducted a series of studies confirming that air filtration can reduce the occurrence of PRRSV infections (30, 31, 83, 84). In laboratory studies, Wenke et al. (85) tested four filters for removing different bioaerosols, including equine arteritis virus (EAV), PRRSV, bovine enterovirus (BEV), *Actinobacillus pleuropneumoniae* (APP), and *Staphylococcus aureus*, and found that air filtration effectively reduces pathogen concentrations. Alonso et al. (29) traced outbreaks of PRRSV in sow farms and concluded that air filtration could significantly reduce the risk of PRRSV aerosol transmission by about 80%. Wenke et al. (86) implemented three different types of filters for existing ventilation systems in pig farms and suggested that recirculating air filter modules resulted in improved pig lung health.

Although there are multiple metrics quantifying the quality of filters in controlled measurement settings (MERV rating), the performance of filters installed on-site still needs further assessment. The installed efficiency in the field varies from that measured in controlled conditions (87); the installed efficiency depends on filter type, airflow (face velocity), the HVAC system, and even the installation as air can bypass the filter if there are gaps at the edge of the filter (87). Furthermore, buildings where air filtration systems are installed may permit some degree of air leakage, given that the building may have construction gaps. Thus, more field tests are needed to better define the effectiveness of filters in farms and provide a better estimate of the cost-to-benefit ratio for the implementation of filters and developing a suitable protocol for maintenance.

### 2.2. Industrially and medically implementable technologies

#### 2.2.1. Electrostatic precipitators

**Figure 5:**
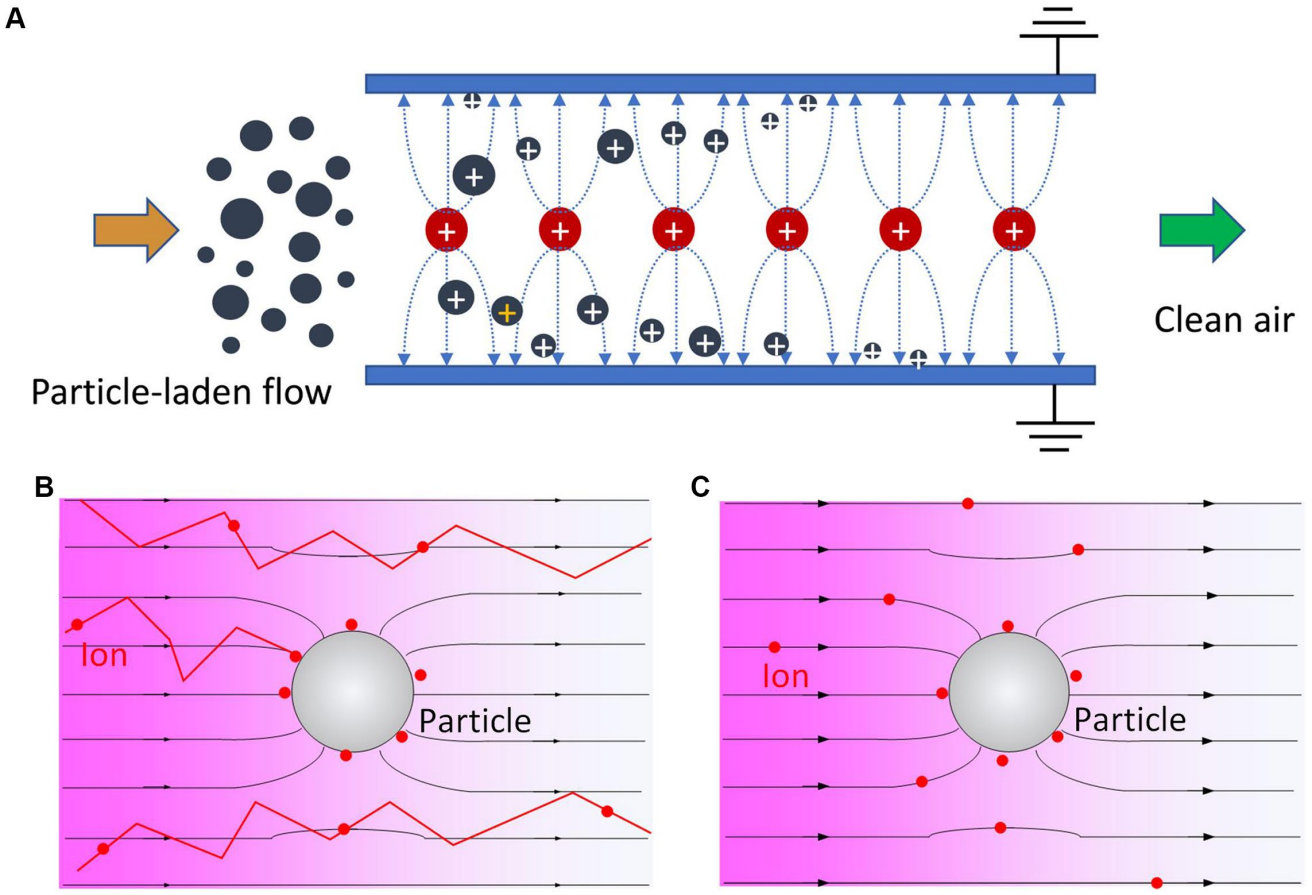
**(A)** Depiction of a wire-plate electrostatic precipitator. A high positive DC voltage is applied to the center wires (red) and the two side plates are grounded. Aerosol particles are charged near the entrance area and collected onto the ground plates. The particle charging mechanisms are depicted in **(B)** (diffusion) and **(C)** (field). The characteristic ion trajectories are represented by red lines.

Electrostatic precipitators (ESPs) are an alternative to filters, primarily used in instances where filter loading and high-pressure drop need to be avoided (often high flow rates with high particle concentrations) (88, 89). An ESP typically consists of two types of electrodes with configurations commonly referred to as wire-plate, wire-cylinder, needle-plate, and spike-plate [see Figure 4 and Table 2 from Qu et al. (90)]. Here, the aerosol flows between both electrodes. A positive or negative high voltage (in the order of several kilovolts) is applied to the wire or needle electrodes, while the plates or cylinder are grounded, resulting in the formation of a corona discharge (91, 92) at the wire or needles (the location of highest electric field intensity due to the small radius of curvature). Ions are generated in a small zone around the high-voltage electrode where the electric field exceeds the critical value for gas breakdown. These ions then move toward the grounded electrodes by Coulomb drift. Positive applied voltage yields positive ions, and negative applied voltage yields negative ions. Aerosol particles enter the ESP with the flow and become charged (93) via random collisions with ions [by a combination of diffusion (94) and field charging mechanisms (95, 96)], thus moving toward the grounded electrodes, provided the residence time in the ESP is sufficient for particle charging and electrophoretic deposition. While diffusion charging (Brownian with Coulomb drift motion, see Figure 5B) is a more relevant charging mechanism of sub-micrometer-sized particles, field charging (charges following electric field lines, see Figure 5C) is commonly the main mechanism of supermicrometer aerosol particle charging. Mainly due to these differences in charging efficiency combined with size-dependent particle electrical mobility, ESPs commonly exhibit a size-dependent collection efficiency with a minimum efficiency in the 0.3–0.6 µm size range and are highly efficient in removing particles below or above this range. The latter has been shown in both experiments and numerical simulations (93). Since the electrodes can vary in configuration, ESPs can be designed in different configurations (e.g., multi-stage, wet or dry, wire-plate, tubular) for various applications. Figure 5A depicts a common wire-plate ESP with positive high voltage applied at the center wires; polydisperse aerosol particles enter the ESP and become positively charged and are collected onto the ground plates at various locations. ESPs, unlike filters, have minimal pressure drop; therefore, they are often well-suited for large-scale applications. For example, high-flow rate (millions of cubic feet per minute) ESPs have been widely used in power plants to remove fly ash, soot, and other pollutants from coal combustion exhaust. (97–99) Gradually, ESPs have also been implemented in indoor air quality control applications, including incorporation in stand-alone recirculating air cleaners and in-duct units in HVAC systems. Afshari et al. (100) have provided a systematic review of ESP as indoor air cleaners.

ESPs have also been used in various embodiments as bioaerosol samplers (101–104) or bioaerosol control devices (105–107). ESPs can control airborne bioaerosol by the collection effects (bioaerosols are collected onto the electrodes) and possibly ionization or reaction effects [viability of bioaerosols can decrease due to ozone, produced in some ESPs, or collision with high-energy ions (93, 108)]. The effectiveness of bioaerosol control is hence a combination of bioaerosol collection and inactivation, which in turn depends upon the particle’s physical efficiency, electric field strength, flow rate, relative humidity, and the design of the ESP. (109) Though a proven technology for particle collection, due to highly variable geometries, lab and field tests are needed to investigate the efficiency of each newly designed ESP device for bioaerosol control application. Also, as ESPs produce ozone (110), therefore, the level of ozone needs to be monitored in each device.

Thus far, to our knowledge, there has been no monitored and reported implementation of ESPs on farms. Nonetheless, like filters, ESPs have the potential to be a usable bioaerosol control technology in farms, because they enable high flow rate without high pressure drop, can often be added to HVAC systems or barn ventilation systems without substantial remodeling, and the electrical power requirements are typically low in comparison to the increase blower power requirements for filters. Furthermore, while filters require regular replacement, ESPs only require electrode cleaning. Cleaning can be obviated as well; wet ESPs (111–113) have been successfully designed and implemented in the power industry, which remove collected particles continuously by a thin water film covering the collection plates. Combining ESPs with filters may also improve the collection efficiency (114) while extending the filter life, thus lowering the cost. In summary, ESP technology is potentially a viable alternative or augmentation to filter systems in farms: both remove bioaerosols by the collection, yet ESP requires less energy due to low-pressure drop. However, ESPs would need to be further tested for such applications.

#### 2.2.2. Ultraviolet light sources

Ultraviolet (UV) light has a shorter wavelength (100–400 nm) compared to visible light and thus has higher energy; it can penetrate into cells, damaging pathogens and acting as a means of disinfection. There are three types of UV sources based on wavelength range: UV-A (315 nm to 400 nm), UV-B (280 nm to 315 nm), and UV-C (100 nm to 280 nm), with energy level inversely proportional to the wavelength. UV-A has less energy and thus is safer for people and livestock; it has been used in activating semiconductor photocatalysts for the mitigation of pathogens (115). UV-C, often referred to as ultraviolet germicidal irradiation (UVGI) (116), has the highest energy among all UV lights. UVGI produced by mercury arc lamps is emitted at 253.7 nm (117), making it extremely effective in damaging nucleic acids (117–120) because the maximum absorption wavelength of nucleic acids occurs near 260 nm.

UVGI has a more than 100-year history in application toward inactivating airborne pathogens (121), and as it is reviewed in detail by Reed (122), we provide only a brief overview here. The effectiveness in inactivation depends on the exposure time and the intensity of irradiation, and hence on system design and operating parameters. UV-C sources can be installed in stand-alone devices in the near-upper region of a room (123) or in HVAC ducts. However, because UVGI acts indiscriminately on nucleic acids, UV-C irradiation exposure poses adverse health effects to humans and livestock, in addition to pathogen inactivation. UVGI should only be in operation when humans and animals are not exposed directly to it, or measures need to be taken so exposure is well below exposure limits. Often, exposure avoidance is accomplished by incorporating UVGI into current HVAC duct systems. The effectiveness of this approach has been demonstrated more than 50 years ago by Jensen (124), where a ducted UV-C source led to greater than 99.9% inactivation for Coxsackie, influenza, Sindbis, and vaccinia viruses, and more than 90% inactivation for a variety of other pathogens. The ducted system in question was also scaled up and installed in the HVAC system of a hospital, with demonstrated as-installed bacteriophage inactivation rates above 99% for flow rates beyond 30,000 m^3^ h^−1^ (125). More recently, in light of the COVID-19 pandemic, Qiao et al. tested a smaller-scale UV-C duct unit and concluded that the unit can achieve greater than 2-log reduction in viable coronavirus inactivation with a flow rate of 2,439 L min^−1^ (126). Since UV-C light itself does not capture or remove pathogens from the fluid stream, it is often used as an additional device to augment current HVAC systems with filters (127); it is commonly available commercially for this purpose. Importantly, during filter disinfection, the filters still collect pathogens and the time of UV-C exposure is increased for collected pathogens, thus the UV-C intensity can be reduced as compared to that needed for standalone ducted systems. Incorporating UV technology into HVAC systems has been recently reviewed by Thornton et al. (128) and by Luo et al. (129). A more recent emerging technology is the far-UV-C (222 nm) source. Though of lower wavelength, because it is farther from 260 nm, it has fewer adverse health effects on humans and animals. Far UV-C application has been demonstrated capable of inactivating airborne pathogens in room-size chambers (130, 131). In addition, as a final note, hand-held UV-C devices typically are not effective in air disinfection. As they can lead to human exposure to UV-C, they are typically of much lower fluence than in-duct units, and hence require significantly longer times for pathogen inactivation. Furthermore, their “coverage” areas are limited, also reducing efficacy.

UV irradiation systems are certainly promising in farms for bioaerosol control due to their high effectiveness in inactivation and ease of implementation. UV-C disinfection can also be used on surfaces (132) while transporting livestock, lowering the risk of introducing viruses during transport. (133) Unlike filters, the implementation of UV-C can also mitigate odor and other gaseous emissions by driving chemical reactions of such species, potentially improving overall air quality on farms and the lung health of animals (134–136). Eisenloffel et al. (137) added a recirculating UV-C module to a barn where air flowed first through a filter module and then passed into a ducted UV-C module. The module effectively reduced bioaerosol concentration in the barn but notably with variable concentrations and levels of effectiveness at different locations, specifically near the exit of the recirculating module; the sampled bioaerosol concentration was much lower than elsewhere. Overall, with high inactivation rates (>99%) UV-C may be installed in the air ductways as a cost-effective alternative to high performance filters in farm-scale applications (138). However, there is presently no information on the use of UV-C in farms on the long-term reduction of disease incidence and the overall functionality of UV-C systems under farm conditions; field testing of UV devices is rare. A final note on the implementation of UV-C for disinfecting air is that measurement of the UV dose is required at the beginning and during operation periodically to make sure the light bulbs are installed correctly and functioning properly (129, 139).

### 2.3. Emerging technologies

Filtration, ESPs, and UV sources are all either actively used in mitigating bioaerosols in farms, or have been scaled to the size and flow rates needed for implementation. At the same time, there are numerous emerging technologies that may be potential candidates to minimize bioaerosol concentrations and aerosol-based disease transmission in farms if demonstrated at scale in the future. These include reactive air disinfection technologies (140) such as ionization, microwave source, photocatalytic systems, non-thermal plasma systems, ozone generators (141) and chemical technologies, as well as devices incorporating these technologies in combination with more traditional technologies (e.g., filters). The majority of these technologies remain under development at the laboratory scale, though some have been successfully commercialized. While it is not within the scope of this review to describe all such technologies in detail, here, we briefly overview several technologies and their potential for implementation on farms.

#### 2.3.1. Ionization technologies – bipolar and unipolar

Ionization technologies have been widely used in clean air applications by adding them in-duct to HVAC systems (142–144) or by incorporating them into stand-alone air purifiers (145, 146). Aerosol particles can deposit on walls both in rooms and HVC systems; in the absence of other forces, deposition is primarily due to diffusive motion or sedimentation. However, when particles are ionized, they will respond to electric fields (as in ESPs), and simply ionizing particles may enhance deposition through naturally or inadvertently present electric fields in indoor spaces, as recently shown by Kolarž et al. (147). Ionization technologies have also been demonstrated for bioaerosol control (148), and may additionally inactivate pathogens through the reactions taking place in them. As in ESPs, ionization technologies often consist of a high voltage electrode (usually a needle) with an applied positive or negative voltage, producing a corona discharge in the air. In unipolar ionization a single polarity is used and particles attain only positive or only negative charge. In bipolar ionization, either two distinct electrodes are used of opposite polarities, or an alternating voltage is applied to a single electrode, resulting in the formation of both positive and negative ions, with the particles becoming both positively and negatively charged. It has been shown that placing an ionizer in line with a filter can enhance filtration efficiency (143, 149), and there are studies noting that ionizers can disinfect bioaerosol-contaminated filter surfaces (150–153). However, the efficacies of devices with ionization technologies vary greatly from device to device and still need further investigation. In addition, the performance of corona ionizers is subject to ambient conditions, including temperature and relative humidity, and thus these parameters should be monitored and reported for repeatable testing and comparison with other filtration technologies (154–156). Zeng et al. (157) tested a commercially available in-duct bipolar ionization device both in chamber tests and in the field and concluded that the device had minimal impact on particle removal within the short duct length in its current setting. The performance of ionizers also depends on ventilation conditions such as flow velocity, air temperature, and relative humidity (158, 159). Byproducts may also form during the ionization process, such as volatile organic components (VOCs) and ozone (O_3_) which need to be monitored during operation (157).

#### 2.3.2. Microwave technologies

Microwaves can inactivate pathogens through both thermal (radiation-induced heating) and non-thermal (microwave irradiation) effects (160, 161). Water absorbs microwave irradiation particularly well, leading to rapid heating. Traditionally, microwaves use heating of water to directly destroy microorganisms, making them suitable for disinfecting, particularly waterborne bacteria and fungi. For airborne bacteria and fungi, microwave systems can still facilitate inactivation but it is less clear whether this is due to heating or non-thermal effects. Several studies suggest that the non-thermal effect is dominant in the inactivation of airborne bacteria (160, 162, 163). For airborne viruses, microwaves can also facilitate inactivation via damaging proteins (164), with several studies suggesting that here too the non-thermal effect is dominant in inactivating airborne viruses (164, 165). In implementation, often, the microwave generator will be installed in the walls of a chamber or duct. The effectiveness of disinfection will depend on the microwave power, bioaerosol type, the flow, system volume (exposure time), and relative humidity (161, 163, 166). Although less studied for bioaerosols, overall, microwaves are an established technology, and hence may find application in bioaerosol mitigation, e.g., in inactivating pathogens on filters (167).

#### 2.3.3. Photocatalytic systems

Photocatalytic materials facilitate chemical reactions when exposed to radiation, such as the generation of reactive oxygen species (ROS), or toxic metal ions if the photocatalytic material contains metal. (168) Such species, in turn, can inactivate pathogens through chemical oxidation reactions, toxic exposure, and even physical damage (169). Photocatalytic disinfection has been applied to waterborne and foodborne viruses in numerous instances (170, 171) and is more established in these applications. However, since the pathogens need to have direct contact with the photocatalytic material to achieve disinfection, or the species generated need to be transported to pathogens, there are significant challenges in adapting photocatalysis alone for airborne pathogens (169).

Most photocatalytic systems are hence coupled with filters to collect bioaerosol particles, with the photocatalytic reaction occurring for collected particles on the filter surface. The most popular photocatalysis material for bioaerosol control is TiO_2_, due to its low cost, nontoxicity, strong oxidizing ability, and long durability (172). Recirculating air purifiers with TiO_2_-based photocatalytic technology have been commercially developed and tested for indoor air quality control including volatile organic components (VOCs) and airborne viruses (173–176). For in-duct HVAC applications, TiO_2_-coated filters or TiO_2_ filters can be utilized for bioaerosol control. (177–179) However, the effectiveness of photocatalysis on disinfection depends on the flow rate, relative humidity, air temperature, catalyst structure, irradiation source, and power (169, 180). The catalyst’s lifetime is limited (181) and requires regeneration or replacement. By-products (182) can also form during oxidation reactions.

#### 2.3.4. Non-thermal plasma (NTP)

Plasmas are a state of matter containing elevated densities of free electrons; such electrons move at extremely high speeds (and hence have high temperatures). However, if the surrounding gas remains at a low temperature, non-equilibrating with the high-speed electrons, it is called a non-thermal plasma (NTP). While NTPs refer broadly to plasmas where the gas is below the electron temperature (and hence the gas may still be elevated in temperature), it is possible to generate the NTP in air where the air temperature remains close to room values. NTPs are highly reactive environments, and hence have been widely investigated for sterilization purposes as they can inactivate pathogens without producing waste. NTP devices have been successfully used to disinfect surfaces and liquid solutions. NTPs have also been used to treat wounds (183), cancer (184), and in a variety of other medical applications (185–187). Several recent review papers (188–192) have been published to examine the current application state of NTP in the decontamination of pathogens, with a focus on viruses (motivated by the COVID-19 pandemic).

With specific regard to airborne pathogens, NTPs generate ions and free electrons, as well as radicals, high-energy photons, and many other reactive oxygen species (ROS) and nitrogen species (RNS), all of which can effectively damage pathogens (188). NTPs can effectively sterilize the air locally (193, 194). Researchers continue to develop NTP technologies for indoor air bioaerosol control. For example, a volumetric dielectric barrier discharge (DBD) (195, 196) non-thermal plasma has been developed toward installation inside an HVAC duct. Nayak et al. demonstrated that an NTP can achieve inactivation of airborne viruses in flight within several milliseconds (197–200). Zhang et al. (201) tested the effects of relative humidity and flow rate for in-duct DBD systems with various electrode arrangements. However, even though multiple studies have demonstrated that DBDs can effectively mitigate viruses in small wind tunnels, field tests are lacking, and further developments are needed to work with large-scale realistic HVAC systems. Scalability remains an issue in NTP application, as does ensuring ROS species are not produced at concentrations too high for humans and animals.

## 3. Testing approaches of technology effectiveness

As noted in the prior section, while there are promising results for a number of emerging bioaerosol control technologies, further testing of such technologies is paramount. Selecting appropriate testing methods for bioaerosol control technologies is challenging, as there are currently no universally applied standards specific to bioaerosols as there are for the characterization of the physical collection efficiency of filters. The choice of testing methods depends largely on the development stage, working principles, and application of the technology. Bioaerosol control technologies generally operate using two principles: inactivation and removal. The testing approaches for technologies that can inactivate pathogens in bioaerosols vary according to their development stage. Typically, small-scale laboratory tests are performed in the early stages of development to demonstrate the technology’s ability to inactivate pathogens on a surface, i.e., after collection. This is often done because of the costs associated with more direct aerosol testing as well as the need for specialized equipment and facilities for aerosol research. Subsequently, chamber or flow tube tests (including larger-scale wind tunnels) are conducted to determine the efficiency of bioaerosol control with tunable operating variables, such as the exposed bioaerosol size distribution, system and control technology flow rate, and relative humidity. Finally, field tests or near-field tests are conducted on-site or in a simulated field environment to evaluate control technology *in-situ* effectiveness for bioaerosol control. In contrast, if the technology’s primary working principle is to collect and remove bioaerosols from the air stream only, small-scale surface inactivation tests are not applicable. Instead, flow tube or chamber tests are more common in initial development, followed by wind tunnel or large room testing. As such devices typically incorporate or function akin to filters, the single-pass wind tunnel, used in ASHRAE testing to determine filter MERV ratings, is ideal for control technologies that couple to the mechanical ventilation systems. Finally, of note is that bioaerosol control devices often integrate multiple technologies to enhance their overall effectiveness. For instance, UV light may be combined with filters to inactivate and remove pathogens from the air in series with one another. Testing may take place on components individually, or on the integrated unit, depending on the state of development.

In this section, we review research relevant to testing control technologies at different scales and different development stages- small-scale testing, including surface inactivation, smaller flow tubes, chambers and wind tunnels, as well as simulated field or true field tests. We then proceed to discuss parameters that must be considered in designing control tests, including bioaerosol generation, sampling, and assays.

### 3.1. Laboratory testing

#### 3.1.1. Small-scale plate test

**Figure 6:**
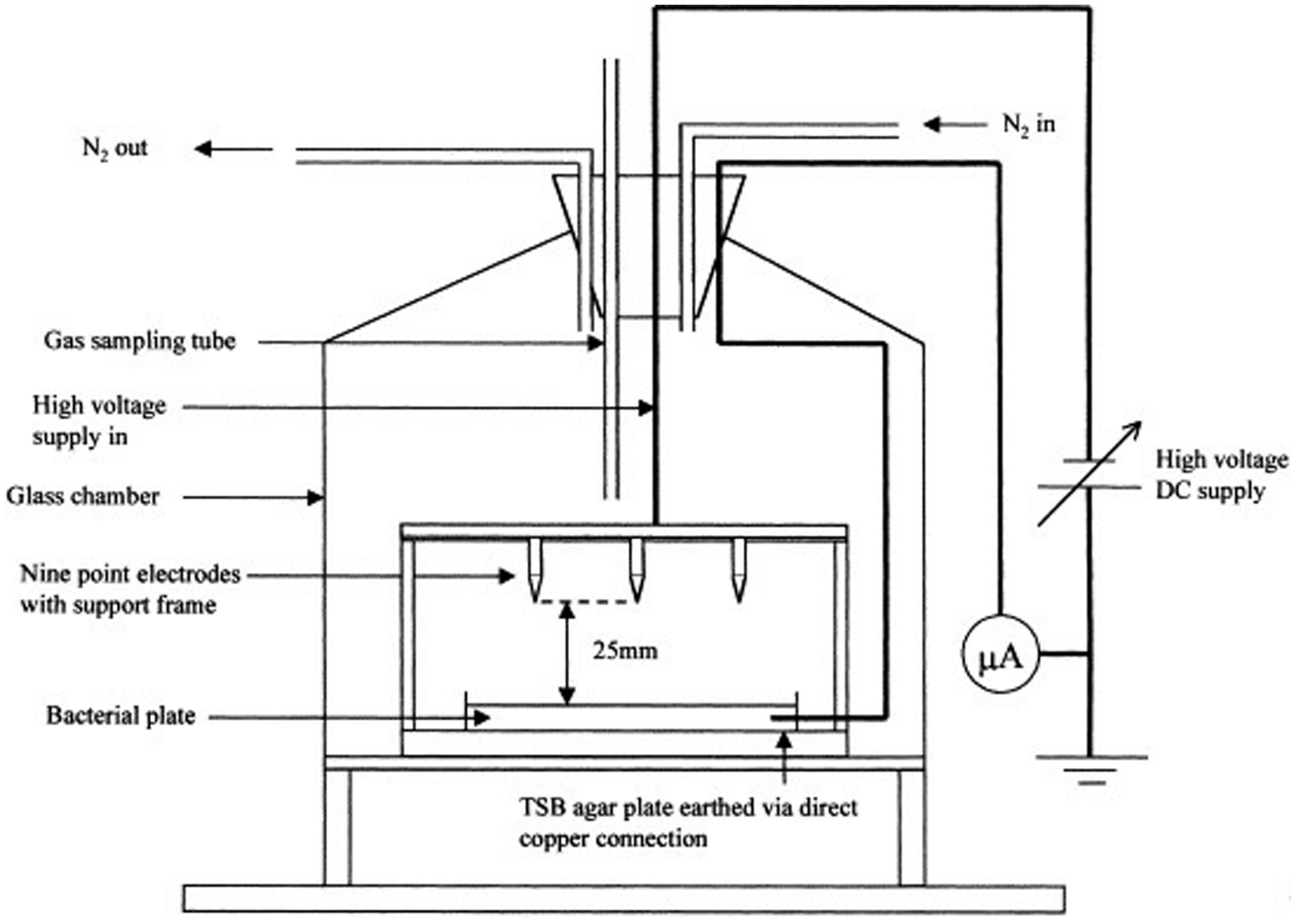
A schematic diagram of an experimental setup for bacterial exposure to negative or positive ions in nitrogen, Adapted from Noyce et al. (204).

Without aerosolizing pathogens, the small-scale plate test often is used to examine the inactivation mechanisms and effectiveness of bioaerosol inactivation technologies over various pathogens, commonly incorporating UV (202, 203), ozone, photocatalysis, non-thermal plasma (200), and ion generation (204) or to demonstrate technologies for surface decontamination applications (205). Figure 6 displays a representative test setup from Noyce et al. (204), used in determining bacterial inactivation rates due to ion bombardment in a high electric field environment. An agar plate with viable pathogens is placed in a biosafety chamber and exposed directly to the inactivation technology. The number of viable pathogens or TCID_50_ (tissue culture infectious dose) is measured with and without the technology after pre-defined exposure times. Because the setup is simple to implement, the test is very useful in earlier technological development stages toward proof-of-concept. However, demonstration of surface inactivation is difficult to extrapolate to performance at large scale and for airborne bioaerosols, as the collection step (if needed) is obviated and the inactivation mechanism acts upon surface-bound pathogens. Therefore, surface tests alone should not be considered complete as validation tests for bioaerosol control technology performance.

#### 3.1.2. Flow-tube tests

**Figure 7:**
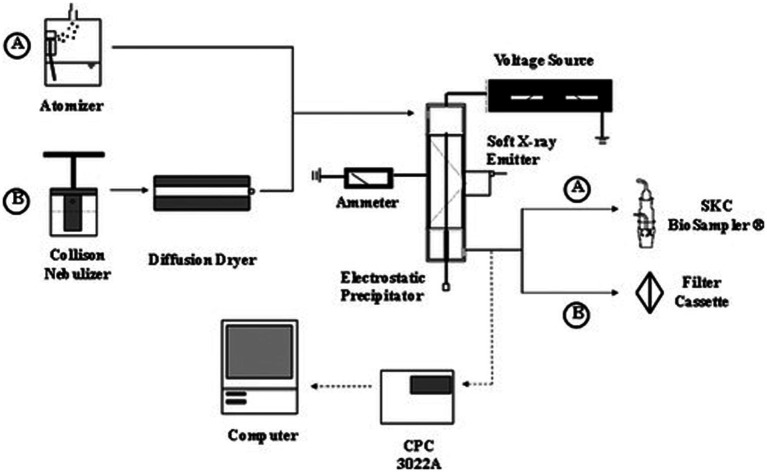
Schematic diagram of an experimental setup for the aerosolization and electrostatic precipitation of bacteriophages T3 (path A) and MS2 (path B). Total particle concentration measurements of the ESP effluent air stream are depicted by dashed lines. Adapted with permission from Kettleson et al. (107).

In addition to the small-scale plate test, tests are needed to demonstrate the inactivation or removal of airborne pathogens. Small-scale flow-tube laboratory tests are likely the most suitable for this purpose. As shown in Figure 7, adapted from Kettleson et al. (107) and used in examining the performance of a soft X-ray enhanced electrostatic precipitator in collecting and inactivating MS2 bacteriophages (107, 206), the test setup typically includes three parts: bioaerosol generation, the in-line technology, and the bioaerosol sampler (for subsequent assay) (119, 124, 194, 207–209). We discuss bioaerosol generation approaches subsequently; in the figure, bioaerosols were generated using an atomizer or nebulizer with a suspension of bacteriophages in culture broth and passed through the technology (ESP in the figure) in operation with control over the volumetric flow rate of air passing through the ESP. Bioaerosols will be either collected (with possible inactivation), not collected but inactivated, or both not collected and not inactivated during passage through the technology. Downstream, the pathogen-containing particles are collected using a sampler, a liquid impinger (BioSampler), and a filter in the diagram, with samples extracted for titration and RT-qPCR (reverse transcriptase quantitative polymerase chain reaction) assays. While flow-tube tests can be run in principle with any flow rate, it is commonplace to first use low or medium flow rates (1–200 L min^−1^) to demonstrate the working mechanism of the technology. Frequently, this leads to laminar flow within the device, while during actual application at scale, flows are turbulent. Smaller flow tubes can also permit larger concentrations of reactive species (e.g., ozone, ions, radicals) than are achievable at a larger scale. Therefore, similar to the surface test, flow-tube tests are important in terms of proof-of-concept and technology development, but should not be extrapolated to larger systems without further testing or without carefully ensuring the conditions tested do not bias results. Large-scale wind tunnel or chamber tests are hence needed to examine technologies with controlled settings (flow rate, relative humidity, room volume) that are closer to application conditions in terms of flow behavior.

#### 3.1.3. Large-scale wind tunnel test

**Figure 8:**
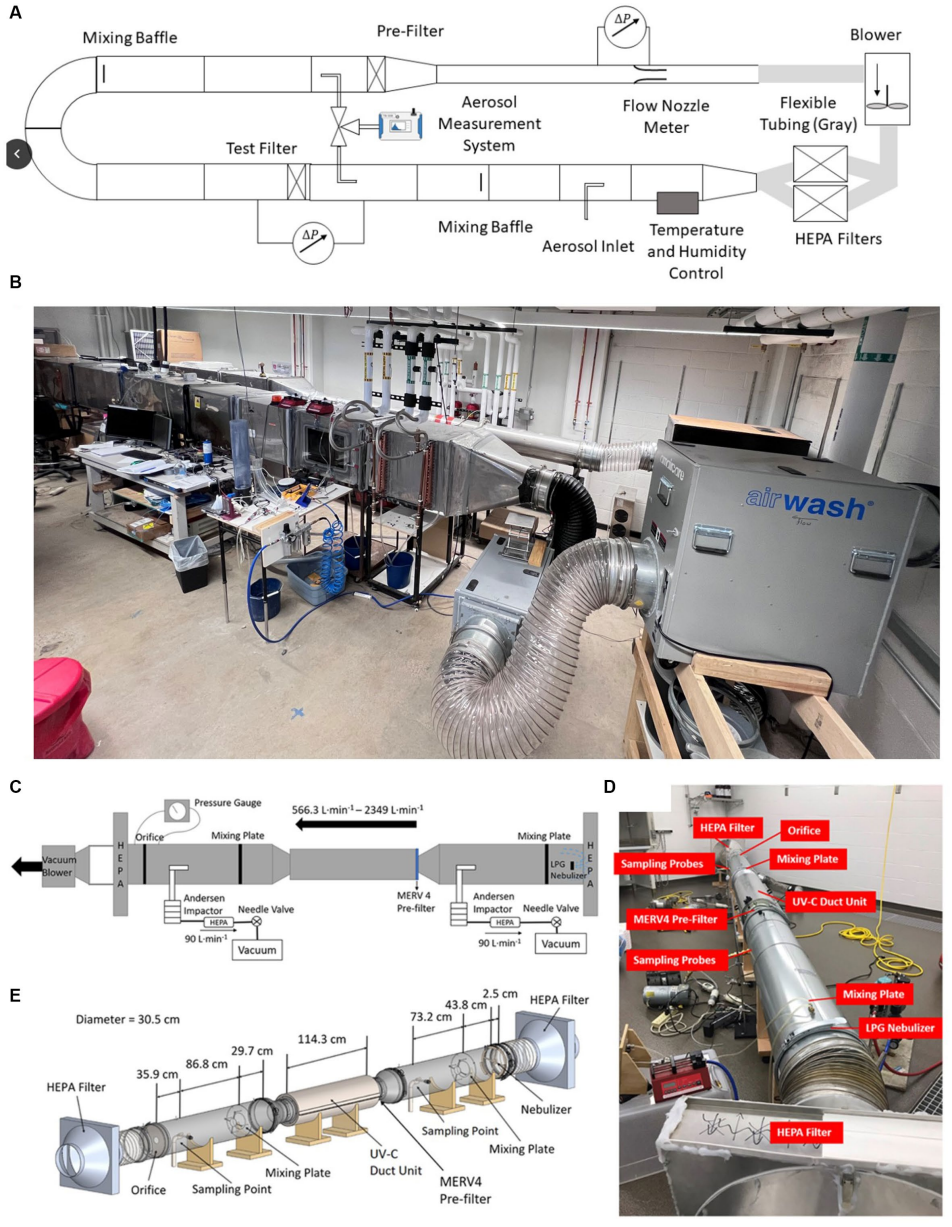
Schematic diagram of an intermediate scale wind tunnel **(A–C)** (126) and regular single-pass wind tunnel **(D,E)** used for testing of bioaerosol collection efficiency of bioaerosol control technologies. The photographs correspond to systems at the University of Minnesota.

Toward implementation, bioaerosol control technologies need to be designed to operate with high flow rates relative to those used in laboratory tests, namely in excess of 10^3^ L min^−1^ and exceeding 10^5^ L min^−1^ for high flow rate HVAC applications. A single-pass wind tunnel test, adapted from ANSI/ASHRAE 52.2 standard, is suitable for both high-flow in-duct technologies as well as recirculating technologies if the inlet and outlet of air can be separated and can operate in this flow rate range. Figures 8A,B depicts a recirculating wind tunnel capable of operating at a flow rate of up to 56,000 L min^−1^ toward use in the ANSI/ASHRAE 52.2 standard test. Such wind tunnels can be used in bioaerosol testing as well; for example, Farnsworth et al. (210) demonstrated the feasibility of using this wind tunnel for filter tests directly with bacterial aerosols, collecting particles with BioSamplers upstream and downstream of the tested filter, as well as extracting particles for assay from the filter (211, 212).

Wind tunnel tests, however, can also be designed for “intermediate scale testing,” i.e., beyond what is commonplace in flow tube systems, but slightly below flow rates encountered in the application (~280 L min^−1^ to 8,000 L min^−1^) (195, 196, 201). It can be necessary to choose intermediate flow rates because of the large costs and safety concerns associated with bioaerosol testing with specific pathogens. For example, Qiao et al. (126, 176) and Ouyang et al. (213) describe an intermediate-scale wind tunnel (depicted in Figures 8C–E) that can be housed within an isolation facility for biosafety level class 2 testing with both alpha- and beta-coronaviruses. As described in Ouyang et al., the flow profiles in these wind tunnels can be controlled to be uniform, as in filter testing, and they enable the installation of both in-duct and recirculating control technologies. While larger in scale, humidity control is nonetheless also possible, as is simultaneous upstream and downstream bioaerosol collection. We do remark that testing at the intermediate wind tunnel scale is less common than surface or smaller flow tube tests. However, because conditions can better match field conditions, we do argue it is an appropriate part of control technology development prior to field testing.

#### 3.1.4. Chamber tests

**Figure 9:**
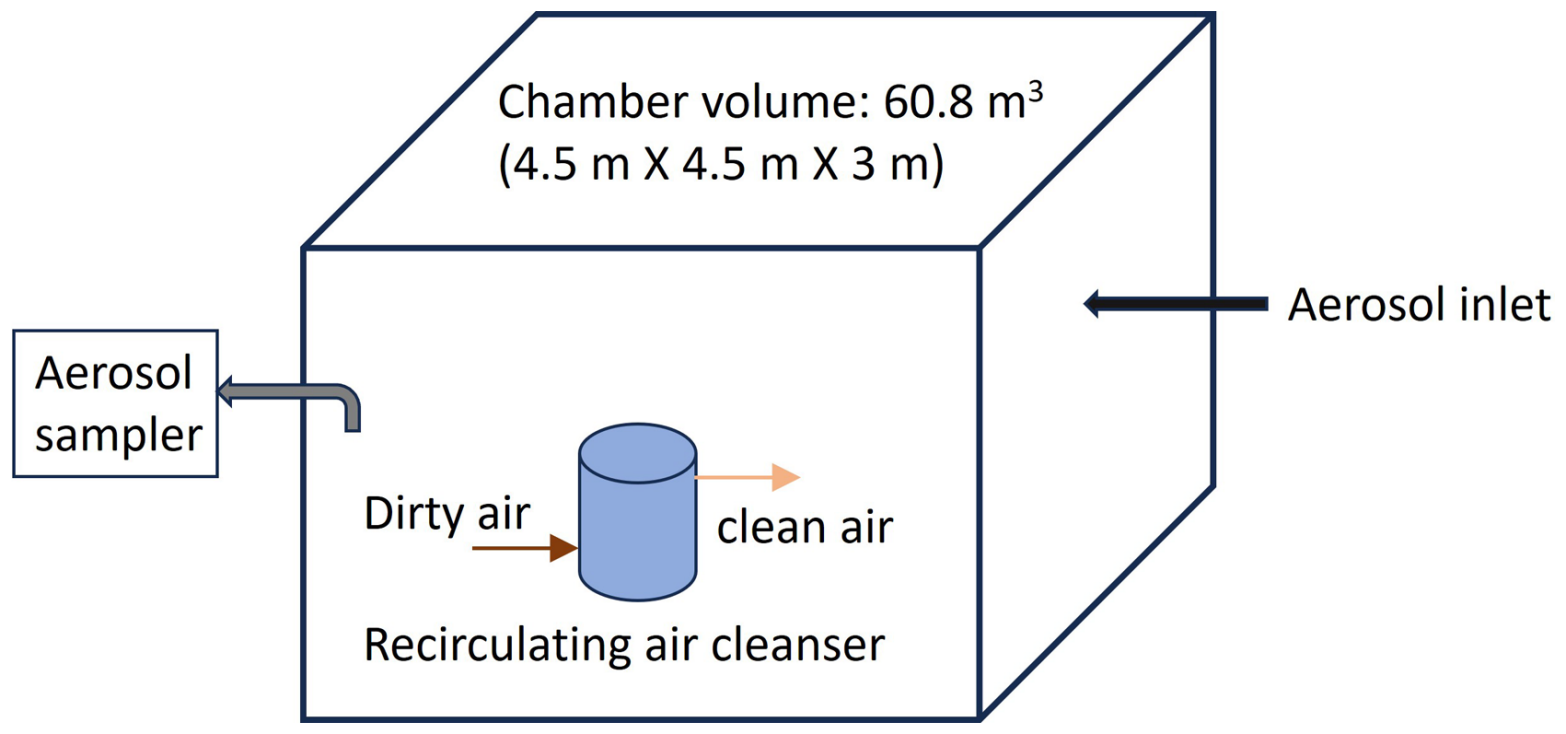
Schematic diagram of a chamber test for a recirculating air cleaner. The chamber volume noted corresponds to the chamber dimensions for the AHAM/ANSI AC-1-2020 test “Method For Measuring Performance of Portable Household Electric Room Air Cleaners”.

A standard chamber has been developed to assess the CADR (Clean Air Delivery Rate) as part of the Association of Home Application Manufacturers/American National Standards Institute (AHAM/ANSI) testing method (214), used largely for the testing of recirculating air cleaners. While the test is not specific to bioaerosols, in it, three different test particles (tobacco smoke, pollen, or dust) are introduced into a sealed room with the size shown in Figure 9, and particle concentration decay is measured with an appropriate real-time particle counter with and without the operation of the air cleaner device. To achieve bioaerosol control, new types of recirculating air cleaners are continuously being developed and tested with a similar procedure to the CADR test, but modified for bioaerosol sampling rather than continuous monitoring of particle concentration (215). In such tests, the chamber is free of bioaerosol at the beginning. Bioaerosol is then delivered to or generated inside the chamber without the operation of the test technology. Right before the technology starts to operate, bioaerosol is sampled from the chamber for the initial concentration assay. The technology starts to operate for a period of time. During operation, bioaerosol continues to be sampled at pre-defined intervals of time for assay. The bioaerosol removal rate is reported as a function of time (216–218) and compared to a control test where the same procedure is repeated without the control technology. Such tests are particularly useful in testing upper-room UV devices (215, 217), as they do not need to be confined in a duct HVAC system in order to operate. The chamber test is straightforward from the adaptation of the CADR test for technologies built upon recirculating air, which can utilize small chambers up to entire room-scale environments, and serves as a near-field test, particularly for upper-room mounted UV devices. However, there is a loss of control of experimental variables in chamber tests in comparison to flow tube and wind tunnel tests. The actual performance of any control technology will be affected by room dimensions and geometry, ventilation system layout (or whether the chamber is sealed), control technology mounting, and even the arrangement of furniture (219). The chamber test is also affected by chamber design, hence greater inter-laboratory variability is expected than in wind tunnel tests. Also noteworthy, analysis in the chamber test implicitly assumes that the aerosol is “well-mixed,” i.e., its spatial concentration variability minimally affects results. It is challenging to keep generated aerosol well-mixed in such tests.

### 3.2. Near-field chamber and field testing

**Figure 10:**
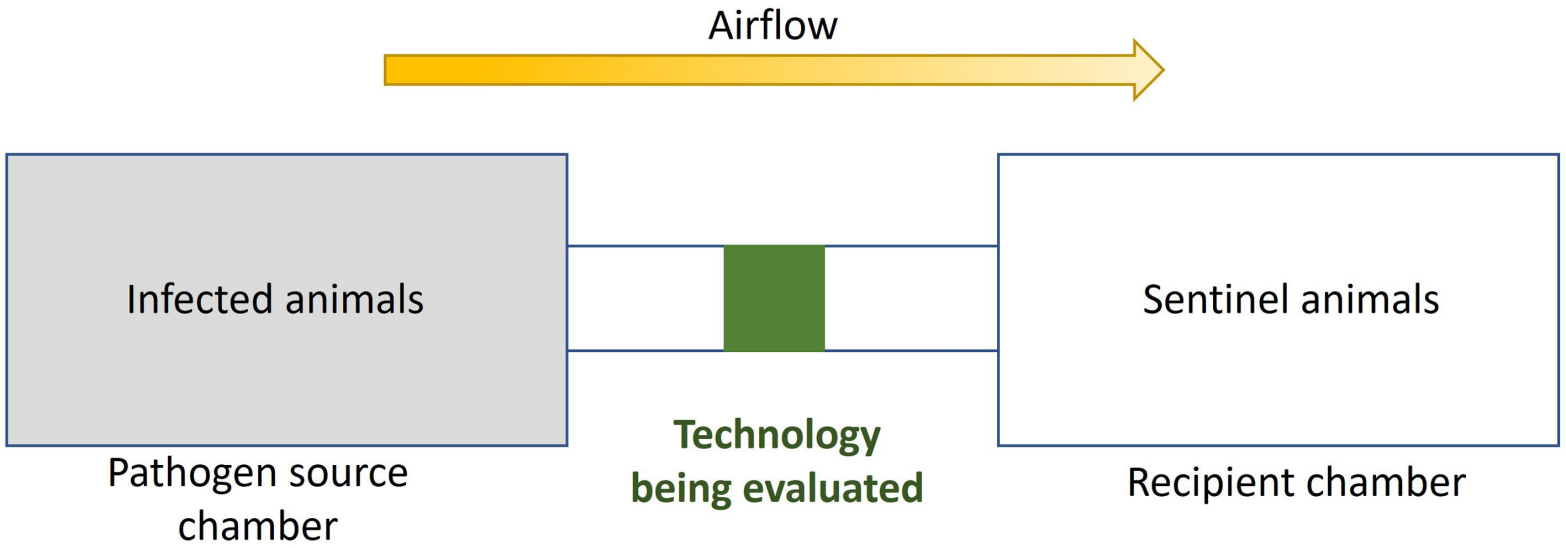
Diagram of experimental near-field chamber testing. Figure with modification from Torremorell et al. (222).

Used more sparingly because of the time, safety level, and cost required, near-field chamber testing (84, 85, 220–222) has been set up to examine the effectiveness of control technologies in preventing airborne pathogens transmission between animals. As shown in Figure 10, near-field chamber tests often have three components: a pathogen source chamber, the control technology (connecting two chambers), and the recipient chamber. Chambers are mechanically ventilated and air flows from the source chamber (upstream of the control technology), passing through the control technology, and exiting to the receiving chamber (downstream of the control technology). There are typically two kinds of experiments, those with and those without animals in the chambers. Without animals in the chamber, pathogens are aerosolized with specific aerosolization devices, similar to flow tube tests and bioaerosols are sampled in both chambers with and without the working control technology under various conditions, such as different flow rates. The collected bioaerosol concentrations are then compared to infer the effectiveness of the control technology. To understand control technology influences on bioaerosol-based transmission, animals (termed sentinels) have been placed in the receiving chamber only (84, 220) or in both chambers. When animals are in both chambers, the source chamber will host infectious seeder animals while the receiving chamber will host naive sentinel animals. Bioaerosols are periodically sampled (typically multiple times over multiple days). At the same time, samples representing body fluids (e.g., blood, saliva, nasal secretions, etc.) are collected to monitor whether the animals are infected with the pathogen. As an example of this type of test, Dee et al. (223) conducted a near-field test for filters as the control technology and reported that all pigs in a chamber downstream of a filter were free of pathogen infection while 30% of the pigs in the no-filter chamber were infected during the experimental period, with PRRS virus as the tested pathogen.

To fully evaluate the benefits and performance of technology and to anticipate issues related to durability and how different factors interact together, technologies do need to be tested under field conditions. There are no specific guidelines on how to evaluate the performance of technologies under field conditions. Measurements not only related to the proper functioning of the technology but also the impact of the technology on decreasing disease incidence and/or prevalence are desirable. Unfortunately, such studies need to be carried out over long periods of time, and hence are difficult to conduct, and costly. Nonetheless, they ultimately provide a more comprehensive assessment of the technology. Such studies allow the identification of other components of using the technology besides the technology itself that are critical to the long-term benefit of using the technology. For example, as noted in prior sections, the installed efficiency of filters can be much lower than expected for a filter’s MERV rating because of bypass flow, arising from poor sealing and leaks during installation. The implications of this may only become clear during a field test.

**Figure 11:**
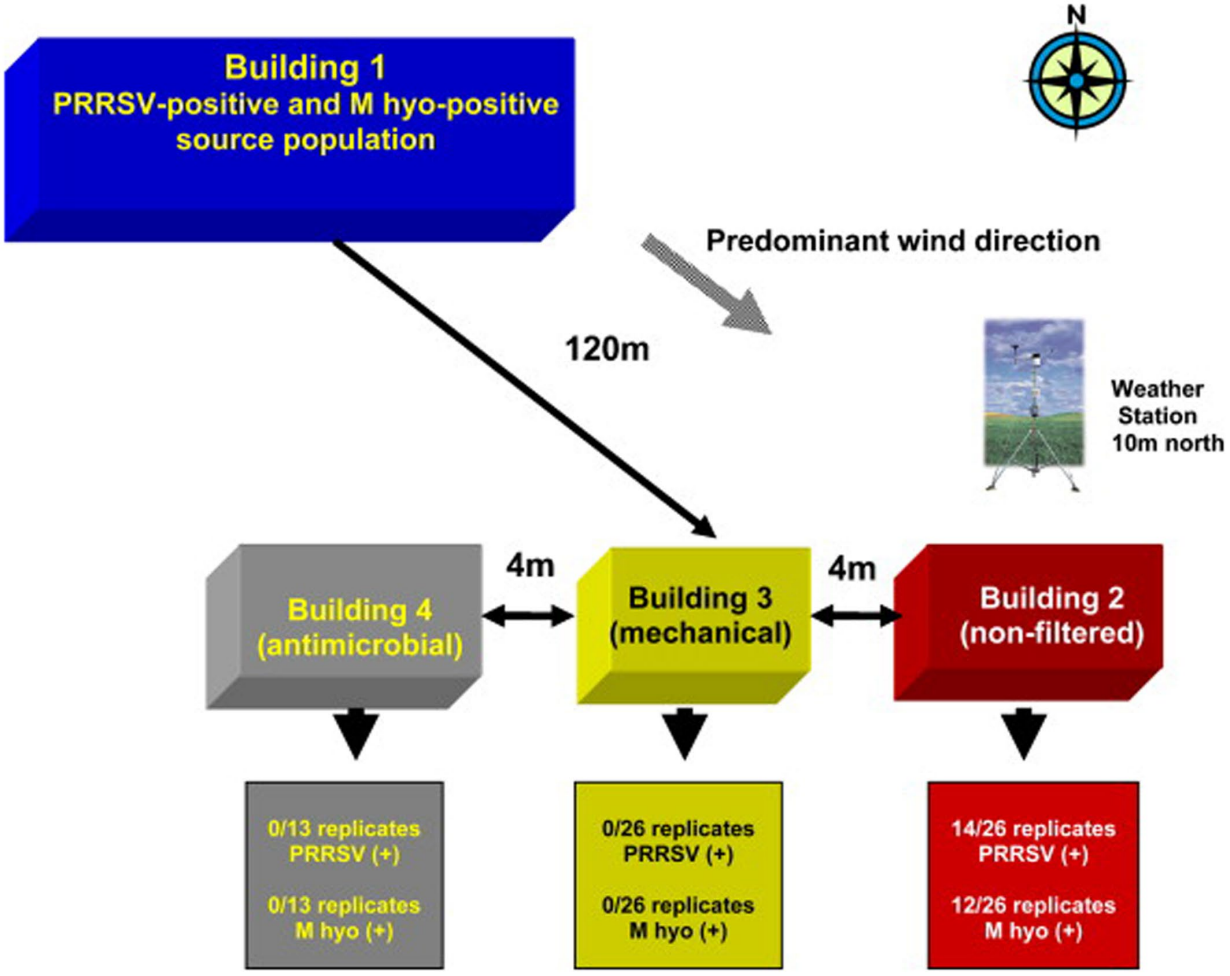
Diagram and representation of a field study building layout where infections were monitored over multiple years. Buildings 1, 2, and 3 were used throughout the 2-year study period while building 4 was only used during year 2. Building 1 served as the source of PRRSV and *Mycoplasma hyopneumoniae* (M hyo)-positive bioaerosols. Buildings 2 (non-filtered control), 3 (mechanical filtration) and 4 (antimicrobial filtration) were placed 120 m downwind to enhance their exposure to bioaerosols transported via prevailing winds. Figure adapted from Dee et al. (83).

Examples of comprehensive field testing using air filtration in swine farms are the studies by Dee et al. (31, 83, 84). Figure 11 depicts a field test configuration where building 1 is about 120 meters from other downwind buildings and hosts 300 pigs as a source of pathogens. The filtered buildings (mechanical and antimicrobial filtration) were free of infected pigs and conversely, about 50% of the pigs were infected in the nonfiltered building. Further in this study, a production system model was designed with infected animals and buildings retrofitted with and without filtration technologies, allowing direct measurements of disease incidence while controlling for other routes of disease transmission. The results from this particular study represented a turning point for the swine industry, highlighting the importance of field study results. The study showed not only that air filtration could work in swine farms but also spoke of the benefits of disease reduction due to the technology. Field testing analysis has continued with the ongoing monitoring of farms and disease incidence against PRRSV. Such studies have provided additional cost–benefit analysis to assist in the decision-making process (28, 29, 33, 224). Field testing has also been helpful in identifying areas of improvement outside of filtration itself but related to using filtration. For instance, in the case of air filtration in swine farms, field testing was necessary to fully identify new air leakage happening at the farm, the durability of construction components, and the impact of the pressure drop as a result of using air filters, which in turn required the development of standard operating procedures and protocols to ensure the technology would work long term.

It is also worth mentioning that testing equivalent to field testing has also been performed for bioaerosol control technologies for humans. In these instances, it is largely through bioaerosol and infection concentration sampling (225) or infection rate monitoring (226) in healthcare settings (227), with and without the presence of tested control technologies. Such studies are time-consuming, and because of the limited ability to control study variables, and to eliminate other routes of transmission, it is often difficult to draw clear conclusions with regards to reduction in incidence of infection. Viable pathogen bioaerosol sampling also remains a challenge, as concentrations are typically low, and viability decays over time for collected pathogens.

### 3.3. Designing test protocols

In all airborne bioaerosol tests, except for small-scale surface tests, biological particles are collected from an aerosol. In laboratory testing, assuming researchers are intentionally producing surrogate pathogen particles, they have control over both the bioaerosol size distribution and the method of collection. Not only are there no standard procedures on control technology performance testing for bioaerosols, but also there are no standardized bioaerosol generation and collection methods, which makes it harder to compare testing results across research groups. Those interested in testing bioaerosol control technologies and in understanding test results need to judiciously evaluate how the results are affected by the bioaerosol generation method and sampling method. In terms of generation, there are two prevailing methods: suspension atomization/nebulization, and fluidization of solid particles. The more commonly employed former method involves utilizing a continuous output, pneumatic nebulizer (often a commercial Collison (228) or BLAM nebulizer) to aerosolize a suspension of the pathogens of interest. Aerosolization has a crucial effect on test results. First, each nebulizer will produce a specific size distribution of particles, affected by the nebulizer geometry and operating conditions (backing pressure) and also the composition of the nebulized media (229). Pathogens are typically incorporated into particles of all sizes generated, and tend to follow the volumetric size distribution unless aerosolization itself leads to size-specific inactivation. It is common for the media to contain solutes that promote pathogen viability in solution. It is common for such solutes to lead to “foaming” during nebulizer operation, which needs to be addressed by adding anti-foaming agents (230, 231). After generation, it is often necessary to charge neutralize the aerosol to mitigate the influence of high charge levels attainable during spray-based aerosol generation (232). It is also necessary to dry the generated droplets, which can be accomplished with driers in smaller systems, but requires time in larger wind tunnels. The nebulizer operating conditions, anti-foaming agents, and drying, may all influence generated pathogen aerosol viability, though there are limited studies directly examining these effects, given the wide variability of possible choices in aerosolization. Recently, it has been shown that the size distribution of virus-carrying particles (with bovine coronavirus as a model system) can be tuned via nebulization to mimic the size distribution of particles measured from human respiratory activities (213, 233), demonstrating that the bioaerosol size distribution can and should be controlled in testing, and then reported as part of testing.

**Figure 12:**
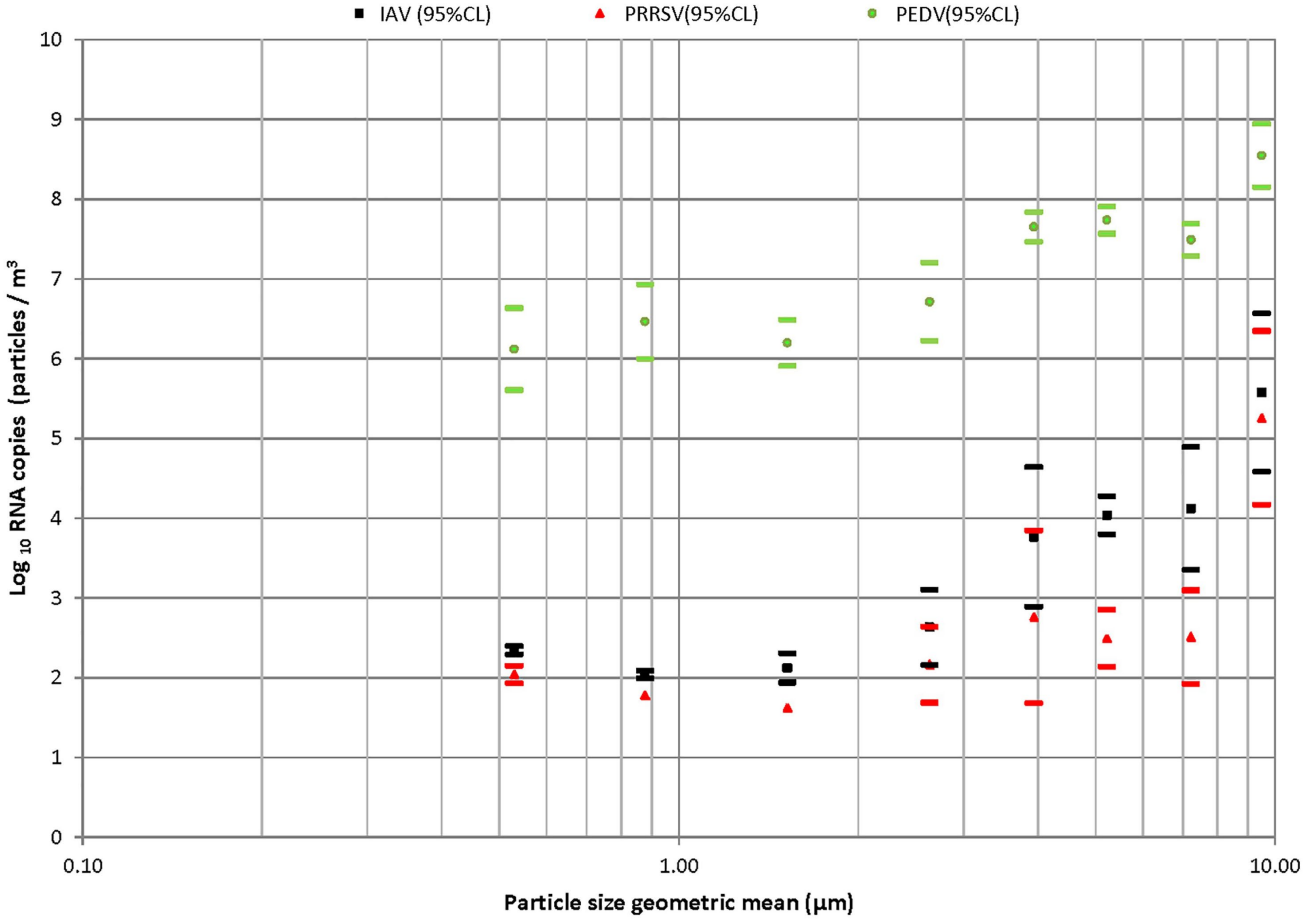
Particle size distributions measured by size-dependent collection device (cascade impaction) and RT-qPCR for influenza, porcine reproductive, and respiratory syndrome and porcine epidemic diarrhea viruses for aerosols generated by infected pigs. Figure adapted from Alonso et al. (248).

As a final note in aerosolization, it is typically desirable to nebulize suspensions with as high a titer as possible for each pathogen, in order to improve signal-to-noise in downstream sampling. Practitioners should make efforts to calculate the pathogen aerosolization rate, i.e., the product of the nebulized suspension titer and the nebulizer liquid feed rate. The input pathogen aerosol concentration is the ratio of this feed rate to the operating air flow rate. This concentration then needs to be considered in sampling, where the upper limit in sampled pathogens is the product of the input pathogen concentration, the sampler flow rate, and the sampling time. Common bioaerosol sampling devices, used to collect bioaerosols from the air, include filters, impactors, impingers, wet-wall cyclones, and electrostatic precipitators. Numerous reviews on bioaerosol sampling, including both active and passive samplers, are available (234–244). We therefore elect not to discuss samplers in greater detail, as collectively such reviews discuss a wide range of sampling approaches. Overall, however, it is important to note that the collection efficiency and biological function recovery efficiency vary among sampling methods (245) due to different sampling mechanisms (206, 246), sampling flow rates, pathogens (245), bioaerosol size distributions, sampling period, and environmental conditions (247) such as relative humidity and temperature. For example, Figure 12 notes the variation in RNA copies as a function of particle size emitted by infected pigs. A sampler with a strongly size-dependent collection efficiency might bring bias or error to the measurements if used in assessing pathogen concentrations from swine. Well-characterized samplers (with known collection efficiencies) used with clearly reported operating conditions are needed when testing control technologies such as to not bias results (for example, through a poor collection of particles larger than 10 micrometers in diameter).

Once collected, follow-up genetic (i.e., RNA/DNA) or viability (the ability of the pathogen to replicate) analysis is performed to evaluate the effectiveness of technologies to remove and/or inactivate pathogens. Here too, multiple reviews have been published on these methods (236, 240, 249). Genetic material tests sampling with and without, or sampling before and after the technology, can reveal the bioaerosol removal efficiency, and viability tests can provide a combined efficiency resulting from both removal and inactivation. Polymerase chain reaction (PCR) assays (250) are the most common methods to detect a pathogen’s genetic material (DNA/RNA). PCR assays detect the genetic material of both viable and non-viable organisms. Viability tests are often culture-based methods for viruses (126) and bacteria (251). Typically, the effectiveness of technologies to remove airborne particles should also be reported in addition to the removal/inactivation of pathogens to provide better indoor air quality. To identify the removal efficiency of the technology, total particle concentrations are measured with and without or before and after the technology. Several instruments exist to measure the total particle concentration and size distribution, including optical particle counter (OPC), aerodynamic particle sizer (APS), and scanning mobility particle sizer (SMPS). These instruments can provide particle concentration with respect to different size bins independently of their nature, shape, or viability.

**Figure 13:**
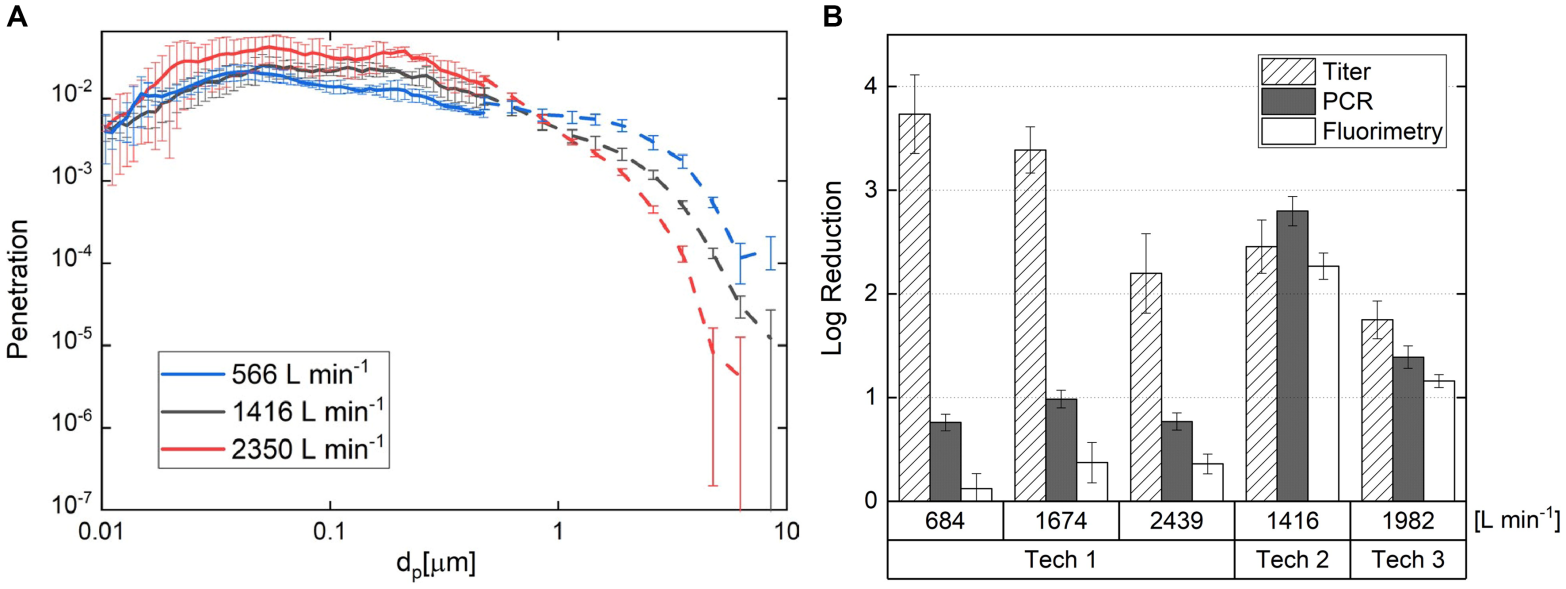
**(A)** Particle penetration versus particle diameter for an air purification unit, measured in a wind tunnel. **(B)** Log reductions of porcine respiratory coronavirus (PRCV) concentration by each of the three tested technologies by virus titration, RT-qPCR, and fluorimetry. Results adapted from Qiao et al. and Ouyang et al. (126, 213).

Finally, the extent of removal or inactivation can be quantified in different manners. Most directly, the collection efficiency is the fraction of pathogens removed through a single pass in the device, which may be determined as a function of particle diameter in more detailed tests. However, for extremely efficient devices, because the difference between collection efficiencies of 99, 99.9, and 99.99% appears small, yet leads to downstream particle and pathogen concentrations that span two orders of magnitude, the penetration or log reduction is often preferred. The penetration is simply equal to (1- collection efficiency), and hence varies by orders of magnitude as the collection efficiency varies from 99 to 99.99%. The log reduction is the base-ten logarithm of the upstream sampled concentration (C_up_) to the downstream concentration (C_down_) in flow tube tests ($\log_{10}\left(C_{\text{up}}/C_{\text{down}}\right)$). Therefore, a log reduction 2 system has a penetration that is ten times lower than a log reduction of 1 system. Note in defining these parameters, we specifically refer to single-pass flow-through systems. In chamber tests, the log reduction is commonly reported as well, but will always increase with time and is often a multi-pass log reduction. We therefore suggest that chamber test results also be converted into single-pass log reductions, to better intercompare results. A method to do this is provided by Ouyang et al. (213). As examples of penetration and log reduction, Figure 13A provides plots of particle size-dependent penetration adapted from Qiao et al. (126), in flow tube tests of a filter-based control technology. Figure 13B displays plots of the log-reduction on physical removal, qRT-PCR, and on virus titration from Ouyang et al. (213), for three different control technologies, all measured in the same flow tube system. Evident from the comparison of log-reduction, technology 1 uniquely has a low log reduction based on physical removal measurements yet a high log-reduction based on virus titer, suggesting it primarily acts through virus inactivation rather than virus removal. Unsurprisingly, this technology is a UV-C based system, while technologies 2 and 3 are filtration systems.

## 4. Implementation and cost analysis

**Figure 14:**
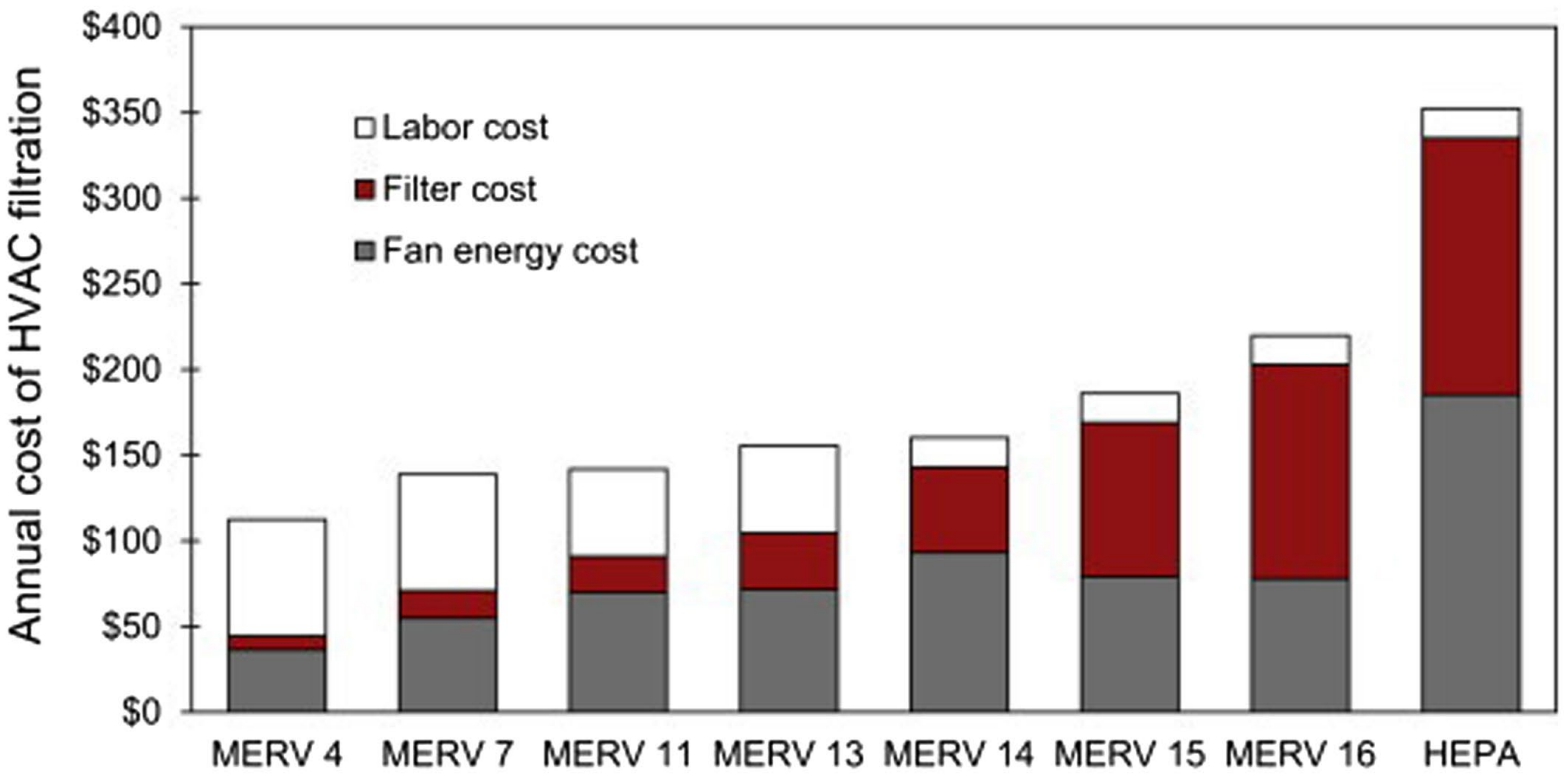
Estimated annual costs of filtration (a single filter) in 2013 in a hypothetical office environment. Adapted from Azimi et al. (70).

It is established that mechanical ventilation systems can be implemented in farms along with control technologies that can prevent farm-to-farm aerosol pathogen transmission and improve animal health (86). For example, the installation of filters into mechanically ventilated farms has successfully prevented PRRSV transmission from infected farms. (19, 28, 252) Nonetheless, filters may be costly due to the number of filters needed, which can be quite large depending on farm size (i.e., from a few hundred to thousands), and typically filters are replaced every three to seven years, and prefilters are replaced every 6–12 months. High-efficiency filters perform better in collecting bioaerosols yet the cost of a single filter increases as performance increases (shown in Figure 14). Filters will also cause an extra pressure drop in the airstream, thus requiring extra power and adding to the energy cost. It is therefore important to consider costs in implementing effective aerosol biosecurity measurements.

Here, we focus on the cost–benefit of implementing control technologies into mechanical ventilation systems, assuming mechanical ventilation systems have already been installed. Specifically, we discuss how to lower the cost of control technologies either by improving or replacing current filtration systems with other technologies to lower the risk due to farm-to-farm aerosol pathogen transmission. The costs and benefits of implementing HVAC filters on residential or commercial buildings to improve indoor air quality have been modeled by several studies (70, 253–258). For human-occupied residential or commercial buildings, the benefit is typically characterized by the modeled reduced risk, and the risk of airborne transmission pathogen is often quantified by a simplified steady-state Wells-Riley (W-R) model (259) where the risk is associated with pathogen generation rates (carrier emitting pathogens), exposure time, the volume of supplied clean air (260), and the infection probability (how many pathogens are needed to successfully infect a person). Here, we mainly focus on preventing airborne transmission of external pathogens from neighboring farms. Thus, we quantify benefits by looking at the total number of collected infectious particles ($N_{i,c}$) and the associated cost with it (C).

To implement bioaerosol control technologies on a farm, several parts of the cost need to be considered, including a fixed cost to purchase and install the technology, a maintenance cost, and an operation cost. The cost of implementing a technology can vary substantially across facilities and factors such as building design, type of construction (new or retrofit), climate, environment (moisture, temperature) and presence of gases (ammonium, hydrogen sulfur, etc.) may affect the longevity and functionality of a particular technology. The operation cost is mainly due to personnel overseeing the ongoing proper functioning of the technology and energy consumption, assuming the system is continuously in operation as turning off or removing the technologies will not protect the animals, which can be represented by the total energy consumed since the final operating cost is equal to the price per energy (dollar/Joule) times the total energy consumed for a given period. While different bioaerosol control technologies have different effectiveness in removing or inactivating bioaerosols, not only the collection efficiency is size dependent, but the quantity of pathogens is also size dependent (261).

Rigorously, for a control technology (e.g., a filter) with a known collection efficiency as a function of particle diameter $CE\left(d_{p}\right)$, exposed over time to aerosol with a size distribution $\frac{dn}{dd_{p}}$ (the number concentration of particles per unit change in diameter) and an effective number of pathogens per particle $\omega \left(d_{p}\right)$ (certainly the greatest unknown in calculations), the number of collected or removed pathogens ($N_{i,c}$) can be calculated as:


(2) $$N_{i,c}=N_{u}{\bar{Q}}_{f}t\int_{0}^{\infty }\frac{dn}{dd_{p}}CE\left(d_{p}\right)\omega \left(d_{p}\right)dd_{p}$$

where N_u_ is the number of bioaerosol control technology units, ${\bar{Q}}_{f}$ is the incoming flow rate per unit, t is the operation time, and d_p_ is the bioaerosol particle diameter. In equation (2), for simplicity, we have assumed that the collection efficiency does not change with time, though as noted in prior sections loading typically leads to collection efficiency changes. The total cost of controlling the incoming airborne pathogens (collection and/or disinfection) is the sum of installation cost, technology purchase cost, energy cost during operation for driving the flow, energy cost during operation for the technology, and maintenance and operating labor cost.


(3) $$\begin{array}{l} C=N_{u}N_{t}c_{\text{inst}}+N_{u}N_{t}c_{\text{tech}}+c_{\text{elec}}N_{u}\frac{\Delta P{\bar{Q}}_{f}t}{\eta_{\text{pump}}}+\\ \;N_{u}IVc_{\text{elec}}t+N_{u}c_{\text{mntc}}t \end{array}$$

where N_t_ is the number of turn-overs (replacement instances), c_inst_ is the one-time installation cost, c_tech_ is the purchase cost, c_elec_ is the electricity cost for providing the flow through the technology, Δ*P* is the pressure drop across the technology (again, assumed constant for simplicity), I is the electric current and V is the electric voltage for operating the technology (also assumed constant), and c_mntc_ is the maintenance labor cost.

Thus the benefit of implementing a control technology is quantified by the ratio of collected/disinfected pathogens over the total cost:


(4) $$\frac{N_{i,c}}{C}=\frac{\int_{0}^{\infty }\frac{dn}{dd_{p}}CE\left(d_{p}\right)\omega \left(d_{p}\right)dd_{p}}{\frac{\left(c_{\text{inst}}+c_{\text{tech}}\right)N_{t}}{{\bar{Q}}_{f}t}+c_{\text{elec}}\frac{\Delta P}{\eta_{\text{pump}}}+\frac{IVc_{\text{elec}}}{{\bar{Q}}_{f}}+\frac{c_{\text{mntc}}}{{\bar{Q}}_{f}}}$$

For simplicity, the integral can be equated with $CEn_{\text{path}}$, where CE is the average collection or removal efficiency over the incoming aerosol size distribution, and $n_{\text{path}}$ is the incoming pathogen concentration in the aerosol (on average). This leads to:


(5) $$\frac{N_{i,c}}{C}=\frac{CEn_{\text{path}}}{\frac{\left(c_{\text{inst}}+c_{\text{tech}}\right)N_{t}}{{\bar{Q}}_{f}t}+c_{\text{elec}}\frac{\Delta P}{\eta_{\text{pump}}}+\frac{IVc_{\text{elec}}}{{\bar{Q}}_{f}}+\frac{c_{\text{mntc}}}{{\bar{Q}}_{f}}}$$

From Equation 5, even without knowledge of the pathogen concentration, individual terms affecting the cost of implementation become clear. To maximize the benefit, technology should be highly efficient on collecting/disinfecting incoming pathogens with a low total cost (summation of the four costs at the denominator). The first installation and technology cost $\left(\frac{\left(c_{\text{inst}}+c_{\text{tech}}\right)N_{t}}{{\bar{Q}}_{f}t}\right)$ could be very high if the replacement is frequent, as in filters; the flow power energy cost $\left(c_{\text{elec}}\frac{\Delta P}{\eta_{\text{pump}}}\;\right)$ will also decrease if the pressure drop decreases, as in UV sources and ESP; the cost to run the electric components of the technology $\frac{IVc_{\text{elec}}}{{\bar{Q}}_{f}}$ could be low for an ESP with low current, but may be higher for UV lights (UV power increases with increasing disinfection efficiency), and is zero for regular filters. Technologies that require minimal operator monitoring and maintenance also become more cost-effective. However, the overwhelming factors reducing cost are to (1) use the highest collection efficiency devices possible and (2) the highest ventilation rates (${\bar{Q}}_{f}$) possible, as these two terms appear in all except the “$c_{\text{elec}}\frac{\Delta P}{\eta_{\text{pump}}}$” term. Stated differently, cost analysis suggests it is beneficial to operate with higher ventilation rates if pathogens are effectively removed, and the pressure drop is not too high.

### 4.1. Cost–benefit analysis considerations from the field

A main outcome of implementing a bioaerosol control technology in animal production is to prevent pathogen entry into a population and cause disease or to reduce disease incidence altogether. Thus, in addition to the aforementioned cost of the technology related to purchase, installation, maintenance and operating costs, the economic feasibility of implementing the technology needs to be measured against the reduction in losses associated with the disease outbreak. Economic losses from a disease outbreak can be measured taking into consideration the performance parameters relevant to the animal species and the production system impacted. Examples of performance parameters to consider include: mortality rates, average daily rate of gain, feed conversion, and number of animals or products sold. Other parameters may include antimicrobial use, additional labor to treat and care for the well-being of the animals, and veterinary cost. The cost/benefit analysis is then measured against the historical frequency of the disease outbreaks caused by the pathogen of interest, the expected severity of the losses and additional costs and compared with the expected parameters should the disease be prevented. An example is the case of PRRS virus infections (224), where the payback period of implementing filtration in swine farms was estimated at 5.35 years or 7.13 years depending on the type of filtration systems used and based on breeding herd productivity but the payback was reduced to 2.1 and 2.8 years based on whether the pigs sold had a premium of $5 to be PRRSV negative. The cost–benefit analysis is often further complicated by the fact that pathogens often spread through multiple routes, making it difficult to know whether the benefit of the technology could not be materialized due to the technology itself or the fact introduction of disease through non-airborne routes could not be prevented.

In summary, the cost–benefit analysis of a given technology should include not only laboratory and modeled data but also a measure of the impact of the technology in preventing disease and productivity losses to production systems.

## 5. Summary

Despite the many biosecurity intervention protocols in farms directed at minimizing the risk of pathogen dissemination through people, animals, and fomites, airborne pathogens can still be transported into, within, and from farms due to the limited use of technologies to mitigate aerosol-based disease transmission. In this report, we review bioaerosol control technologies that can be implemented to prevent airborne transmission of pathogens between farms. Mechanical ventilation systems with filters can remove pathogens from the incoming air and thus have been effective at preventing the spread of the PRRS virus. However, filters are costly and other technologies are hence proposed to either replace or assist filters (prolong the filter’s life) to lower the cost and reach the same or even better decontamination efficiency. At present, ESPs and UVC devices are the most promising technologies for farm applications which can possibly replace and/or complement filters and work with mechanical ventilation systems alone, as they have been proven to work in industrial environments and in healthcare environments, respectively. However, those technologies have not been adapted yet to farms and it is unknown whether their cost/benefit analysis would support their implementation. Other emerging technologies may find application in farms in the future but require additional testing at multiple levels first.

For this reason, this report also focuses on reviewing testing setups for bioaerosol control technologies, in an effort to harmonize testing approaches, as emerging control technologies are often tested inconsistently from one another. We find many testing parameters need to be considered when comparing technology effectiveness, such as the method of bioaerosol generation, collection for analysis, experimental setup (flow tube versus chamber), and in-file performance among others, as they all affect the resulting data.

Lastly, preventing disease introduction into farms is a priority to ensure food security. We note the choice of technologies depends on the specific configuration of the farm, the cost–benefit analysis, and the ability of technologies to operate under specific environmental conditions determined by the farm type. Airborne control technologies should be used in conjunction with all other on-farm biosecurity protocols to comprehensively limit the introduction and spread of pathogens through all possible routes. Furthermore, airborne control technologies should be considered as part of the package of biosecurity measures employed by farmers to prevent disease introduction, in particular for animals housed in confinement in mechanically ventilated buildings with high densities and for farms located in regions where proximity to other farms is a risk. Lastly, to ensure food security, preventing disease introduction into and spread from farms should be a priority thus, further research in aerosol technologies is warranted.

## Author contributions

HO: Investigation, Methodology, Visualization, Writing – original draft, Writing – review & editing. LW: Investigation, Writing – review & editing. DS: Visualization, Writing – review & editing. MY: Writing – review & editing, Investigation. JM: Visualization, Writing – review & editing, Investigation. LL: Writing – review & editing. BO: Writing – review & editing, Conceptualization, Investigation. MS: Writing – review & editing, Writing – original draft. CH: Conceptualization, Methodology, Writing – original draft, Writing – review & editing, Funding acquisition, Investigation, Project administration. MT: Conceptualization, Methodology, Writing – original draft, Writing – review & editing, Funding acquisition, Investigation, Project administration.

## References

[1] Hyslop NS. Factors influencing the epidemiology and epizootiology of airborne diseases. J Am Vet Med Assoc. (1971) 159:1500–7. PMID: 4342868

[2] Wang CC Prather KA Sznitman J Jimenez JL Lakdawala SS Tufekci Z. Airborne transmission of respiratory viruses. Science (1979). (2021) 373:9149. doi: 10.1126/science.abd9149, PMID: 34446582 PMC8721651

[3] Jones RM Brosseau LM. Aerosol transmission of infectious disease. J Occup Environ Med. (2015) 57:501–8. doi: 10.1097/JOM.0000000000000448, PMID: 25816216

[4] Ather B Mirza TM Edemekong PF. Airborne precautions. Treasure Island: StatPearls Publishing (2023). 30285363

[5] Stark KDC. (1999). The role of infectious aerosols in disease transmission in pigs. Available at: http://www.idealibrary.com (Accessed November 2, 2023). 10.1053/tvjl.1998.0346 10558836

[6] Alonso C Goede DP Morrison RB Davies PR Rovira A Marthaler DG. Evidence of infectivity of airborne porcine epidemic diarrhea virus and detection of airborne viral RNA at long distances from infected herds. Vet Res. (2014) 45:73. doi: 10.1186/s13567-014-0073-z, PMID: 25017790 PMC4347589

[7] Wright DN Bailey GD Goldberg LJ. Effect of temperature on survival of airborne *Mycoplasma pneumoniae*. J Bacteriol. (1969) 99:491–5. doi: 10.1128/jb.99.2.491-495.1969, PMID: 5808076 PMC250044

[8] Quinn P Markey B Carter M Donnelly W Leonard F. Veterinary Microbiology and microbial disease. Ames: Iowa State University Press (2002).

[9] Leibler JH Otte J Roland-Holst D Pfeiffer DU Soares Magalhaes R Rushton J. Industrial food animal production and global health risks: exploring the ecosystems and economics of avian influenza. EcoHealth. (2009) 6:58–70. doi: 10.1007/s10393-009-0226-0, PMID: 19437076 PMC7087879

[10] Tilman D Balzer C Hill J Befort BL. Global food demand and the sustainable intensification of agriculture. Proc Natl Acad Sci U S A. (2011) 108:20260–4. doi: 10.1073/pnas.1116437108, PMID: 22106295 PMC3250154

[11] APHIS. Final Report for the 2014-2015 Outbreak of Highly Pathogenic Avian Influenza (HPAI) in the United States Public Version. (2016). Available at: https://www.aphis.usda.gov/animal_health/emergency_management/downloads/hpai/2015-hpai-final-report.pdf (Accessed August 13, 2023).

[12] Holtkamp D Linhares D De Sousa E Silva G Lopez W Corzo C Vilalta C. Monitoring and updating the value of productivity losses due to porcine reproductive and respiratory syndrome virus. Iowa State University. (2018).

[13] World Organization for Animal Health. (n.d.) Porcine reproductive and respiratory syndrome. Available at: https://www.woah.org/en/disease/porcine-reproductive-and-respiratory-syndrome/ (Accessed November 2, 2023).

[14] Abdisa Serbessa T Gemechu Geleta Y Obsa TI. Review on diseases and health management of poultry and swine. Int J Avian Wildlife Biol. (2023) 7:27–38. doi: 10.15406/ijawb.2023.07.00187, PMID: 37196748

[15] Alarcón LV Allepuz A Mateu E. Biosecurity in pig farms: a review. Porcine Health Manag. (2021) 7:5. doi: 10.1186/s40813-020-00181-z, PMID: 33397483 PMC7780598

[16] Otake S Dee S Corzo C Oliveira S Deen J. Long-distance airborne transport of infectious PRRSV and *Mycoplasma hyopneumoniae* from a swine population infected with multiple viral variants. Vet Microbiol. (2010) 145:198–208. doi: 10.1016/j.vetmic.2010.03.028 20418029

[17] Lambert MÈ Arsenault J Poljak Z D’Allaire S. Correlation among genetic, Euclidean, temporal, and herd ownership distances of porcine reproductive and respiratory syndrome virus strains in Quebec, Canada. BMC Vet Res. (2012) 8:76. doi: 10.1186/1746-6148-8-76 22676411 PMC3436738

[18] Dee S Otake S Oliveira S Deen J. Evidence of long distance airborne transport of porcine reproductive and respiratory syndrome virus and *Mycoplasma hyopneumoniae*. Vet Res. (2009) 40:39. doi: 10.1051/vetres/2009022, PMID: 19379664 PMC2701181

[19] Pitkin A Deen J Dee S. Use of a production region model to assess the airborne spread of porcine reproductive and respiratory syndrome virus. Vet Microbiol. (2009) 136:1–7. doi: 10.1016/j.vetmic.2008.10.013 19046835

[20] Donaldson A Gloster J Harvey L Deans D. Use of prediction models to forecast and analyse airborne spread during the foot-and-mouth disease outbreaks in Brittany, Jersey and the Isle of Wight in 1981. Vet Rec. (1982) 110:53–7. doi: 10.1136/vr.110.3.53, PMID: 7064324

[21] Hugh-Jones ME Wright PB. Studies on the 1967–8 foot-and-mouth disease epidemic: the relation of weather to the spread of disease. J Hyg. (1970) 68:253–71. doi: 10.1017/S0022172400028722, PMID: 5270205 PMC2130799

[22] Garten RJ Davis CT Russell CA Shu B Lindstrom S Balish A. Antigenic and genetic characteristics of swine-origin 2009 a(H1N1) influenza viruses circulating in humans. Science. (2009) 325:197–201. doi: 10.1126/science.1176225 19465683 PMC3250984

[23] Corzo CA Allerson M Gramer M Morrison RB Torremorell M. Detection of airborne influenza a virus in experimentally infected pigs with maternally derived antibodies. Transbound Emerg Dis. (2014) 61:28–36. doi: 10.1111/j.1865-1682.2012.01367.x, PMID: 22827737

[24] Corzo CA Culhane M Dee S Morrison RB Torremorell M. Airborne detection and quantification of swine influenza a virus in air samples collected inside, outside and downwind from swine barns. PLoS One. (2013) 8:1444. doi: 10.1371/journal.pone.0071444, PMID: 23951164 PMC3738518

[25] James J Warren CJ De Silva D Lewis T Grace K Reid SM. The role of airborne particles in the epidemiology of clade 2.3.4.4b H5N1 high pathogenicity avian influenza virus in commercial poultry production units. Viruses. (2023) 15:1002. doi: 10.1101/2023.03.16.532935 37112981 PMC10142477

[26] Bertran K Balzli C Kwon YK Tumpey TM Clark A Swayne DE. Airborne transmission of highly pathogenic influenza virus during processing of infected poultry. Emerg Infect Dis. (2017) 23:1806–14. doi: 10.3201/eid2311.170672, PMID: 29047426 PMC5652435

[27] Torremorell M Alonso C Davies PR Raynor PC Patnayak D Torchetti M. Investigation into the airborne dissemination of H5N2 highly pathogenic avian influenza virus during the 2015 spring outbreaks in the midwestern United States. Avian Dis. (2016) 60:637–43. doi: 10.1637/11395-021816-Reg.1, PMID: 27610723

[28] Alonso C Otake S Davies P Dee S. An evaluation of interventions for reducing the risk of PRRSV introduction to filtered farms via retrograde air movement through idle fans. Vet Microbiol. (2012) 157:304–10. doi: 10.1016/j.vetmic.2012.01.010, PMID: 22306035

[29] Alonso C Murtaugh MP Dee SA Davies PR. Epidemiological study of air filtration systems for preventing PRRSV infection in large sow herds. Prev Vet Med. (2013) 112:109–17. doi: 10.1016/j.prevetmed.2013.06.001, PMID: 23870693

[30] Dee S Cano JP Spronk G Reicks D Ruen P Pitkin A. Evaluation of the Long-term effect of air filtration on the occurrence of new PRRSV infections in large breeding herds in swine-dense regions. Viruses. (2012) 4:654–62. doi: 10.3390/v4050654, PMID: 22754642 PMC3386623

[31] Dee S Spronk G Reicks D Ruen P Deen J. Further assessment of air filtration for preventing PRRSV infection in large breeding pig herds. Vet Rec. (2010) 167:976–7. doi: 10.1136/vr.c6788, PMID: 21262714

[32] MSHMP. (n.d.) Morrison swine health monitoring program. PRRS cumulative incidence. Available at: https://mshmp.umn.edu/reports#Charts (Accessed August 11, 2023).

[33] Havas KA Brands L Cochrane R Spronk GD Nerem J Dee SA. An assessment of enhanced biosecurity interventions and their impact on porcine reproductive and respiratory syndrome virus outbreaks within a managed group of farrow-to-wean farms, 2020–2021. Front Vet Sci. (2023) 9:952383. doi: 10.3389/fvets.2022.952383 36713879 PMC9879578

[34] Batista L Pouliot F Dufour V Morin M (2009). How does air filtration fit into porcine reproductive and respiratory virus regional control and eradication strategies?. Available at: https://www.researchgate.net/publication/228446984 (Accessed November 2, 2023).

[35] Yang X Haleem N Osabutey A Cen Z Albert KL Autenrieth D. Particulate matter in swine barns: A comprehensive review. Atmosphere. (2022) 13:490. doi: 10.3390/atmos13030490

[36] Guo L Zhao B Jia Y He F Chen W. Mitigation strategies of air pollutants for mechanical ventilated livestock and poultry housing—A review. Atmosphere. (2022) 13:452. doi: 10.3390/atmos13030452

[37] La A Zhang Q Cicek N Coombs KM. Current understanding of the airborne transmission of important viral animal pathogens in spreading disease. Biosyst Eng. (2022) 224:92–117. doi: 10.1016/j.biosystemseng.2022.09.013

[38] Larriba-Andaluz C Carbone F. The size-mobility relationship of ions, aerosols, and other charged particle matter. J Aerosol Sci. (2021) 151:105659. doi: 10.1016/j.jaerosci.2020.105659

[39] Suárez-Peña B Negral L Castrillón L Megido L Marañón E Fernández-Nava Y. Imaging techniques and scanning electron microscopy as tools for characterizing a Si-based material used in air monitoring applications. Materials. (2016) 9:109. doi: 10.3390/ma9020109, PMID: 28787908 PMC5456495

[40] Kim SC Kang S Lee H Kwak D-B Ou Q Pei C. Nanofiber filter performance improvement: nanofiber layer uniformity and branched nanofiber. Aerosol Air Qual Res. (2020) 20:80–8. doi: 10.4209/aaqr.2019.07.0343

[41] Lou CW Shih YH Huang CH Lee SA Chen YS Lin JH. Filtration efficiency of electret air filters reinforced by titanium dioxide. Appl Sci. (2020) 10:2686. doi: 10.3390/app10082686

[42] Kim S Chung J Lee SH Yoon JH Kweon DH Chung WJ. Tannic acid-functionalized HEPA filter materials for influenza virus capture. Sci Rep. (2021) 11:3487. doi: 10.1038/s41598-021-03487-2, PMID: 33441577 PMC7806633

[43] Bin KH Lee WJ Choi SC Lee KB Lee MH. Filter quality factors of fibrous filters with different fiber diameter. Aerosol Sci Technol. (2021) 55:154–66. doi: 10.1080/02786826.2020.1829535

[44] Hinds WC Zhu Y. (n.d.) Aerosol technology: Properties, behavior, and measurement of airborne particles. Wiley-Interscience; 2nd ed (1999).

[45] Zhu M Han J Wang F Shao W Xiong R Zhang Q. Electrospun nanofibers membranes for effective air filtration. Macromol Materials Eng. (2017) 302:353. doi: 10.1002/mame.201600353

[46] Zhu C Lin CH Cheung CS. Inertial impaction-dominated fibrous filtration with rectangular or cylindrical fibers. Powder Technol. (2000) 112:149–62. doi: 10.1016/S0032-5910(99)00315-0

[47] Hinds WC Zhu Y. Aerosol technology: Properties, behavior, and measurement of airborne particles. Wiley; 3rd ed (2022).

[48] Kirsch AA Chechuev PV. Diffusion deposition of aerosol in fibrous filters at intermediate Peclet numbers. Aerosol Sci Technol. (1985) 4:11–6.

[49] Kirsch AA Fuchs NA. Studies on fibrous aerosol filters—III diffusional deposition of aerosols in fibrous filters. Ann Occup Hyg. (1968). doi: 10.1093/annhyg/11.4.299, PMID: 5721714

[50] Lee KW Liu BYH. Theoretical study of aerosol filtration by fibrous filters. Aerosol Sci Technol. (1982) 1:147–61. doi: 10.1080/02786828208958584

[51] Ramarao BV Tien C Mohan S. Calculation of single fiber efficiencies for interception and impaction with superposed brownian motion. J Aerosol Sci. (1994) 25:295–313. doi: 10.1016/0021-8502(94)90081-7

[52] Lee KW Liu BYH. On the minimum efficiency and the Most penetrating particle size for fibrous filters. J Air Pollut Control Assoc. (1980) 30:377–81. doi: 10.1080/00022470.1980.10464592

[53] Kirsh AA Stechkina IB Fuchs NA. Efficiency of aerosol filters made of ultrafine polydisperse fibres. J Aerosol Sci. (1975) 6:119–24. doi: 10.1016/0021-8502(75)90004-X

[54] Baumgartner HP Löffler F. The collection performance of electret filters in the particle size range 10 nm-10 μm. J Aerosol Sci. (1986) 17:438–45. doi: 10.1016/0021-8502(86)90126-6

[55] Lee M Otani Y Namiki N Emi H. Prediction of collection efficiency of high-performance electret filters. J Chem Eng Japan. (2002) 35:57–62. doi: 10.1252/jcej.35.57

[56] Bai H Qian X Fan J Shi Y Duo Y Guo C. Theoretical model of single fiber efficiency and the effect of microstructure on fibrous filtration performance: a review. Ind Eng Chem Res. (2021) 60:3–36. doi: 10.1021/acs.iecr.0c04400

[57] Tufenkji N Elimelech M. Correlation equation for predicting single-collector efficiency in physicochemical filtration in saturated porous media. Environ Sci Technol. (2004) 38:529–36. doi: 10.1021/es034049r, PMID: 14750730

[58] ASHRAE Standard. ASHRAE Standard 52.2–2017, method of testing general ventilation air-cleaning devices for removal efficiency by particle size. Atlanta: ASHRAE (2017).

[59] United States Environmental Protection Agency (EPA). (n.d.) What is a MERV rating? Available at: https://www.epa.gov/indoor-air-quality-iaq/what-merv-rating (Accessed August 6, 2023).

[60] Podgórski A Bałazy A Gradoń L. Application of nanofibers to improve the filtration efficiency of the most penetrating aerosol particles in fibrous filters. Chem Eng Sci. (2006) 61:6804–15. doi: 10.1016/j.ces.2006.07.022

[61] Huang SH Chen CW Kuo YM Lai CY McKay R Chen CC. Factors affecting filter penetration and quality factor of particulate respirators. Aerosol Air Qual Res. (2013) 13:162–71. doi: 10.4209/aaqr.2012.07.0179

[62] Grillet AM Nemer MB Storch S Sanchez AL Piekos ES Leonard J. COVID-19 global pandemic planning: performance and electret charge of N95 respirators after recommended decontamination methods. Exp Biol Med. (2021) 246:740–8. doi: 10.1177/1535370220976386, PMID: 33325749 PMC7961645

[63] Yim W Cheng D Patel SH Kou R Meng YS Jokerst JV. KN95 and N95 respirators retain filtration efficiency despite a loss of dipole charge during decontamination. ACS Appl Mater Interfaces. (2020) 12:54473–80. doi: 10.1021/acsami.0c17333, PMID: 33253527 PMC7724761

[64] Chen DR Pui DYH Liu BYH. Optimization of pleated filter designs using a finite-element numerical model. Aerosol Sci Technol. (1995) 23:579–90. doi: 10.1080/02786829508965339

[65] Fotovati S Hosseini SA Vahedi Tafreshi H Pourdeyhimi B. Modeling instantaneous pressure drop of pleated thin filter media during dust loading. Chem Eng Sci. (2011) 66:4036–46. doi: 10.1016/j.ces.2011.05.038

[66] Hanley JT Ensor DS Smith DD Sparks LE. Fractional aerosol filtration efficiency of in-duct ventilation air cleaners. Indoor Air. (1994) 4:169–78. doi: 10.1111/j.1600-0668.1994.t01-1-00005.x

[67] Miaskiewicz-Peska E Lebkowska M. Comparison of aerosol and bioaerosol collection on air filters. Aerobiologia (Bologna). (2012) 28:185–93. doi: 10.1007/s10453-011-9223-1, PMID: 22523449 PMC3321141

[68] Srikrishna D. Can 10× cheaper, lower-efficiency particulate air filters and box fans complement high-efficiency particulate air (HEPA) purifiers to help control the COVID-19 pandemic? Sci Total Environ. (2022) 838:155884. doi: 10.1016/j.scitotenv.2022.155884, PMID: 35580674 PMC9107182

[69] Pease LF Wang N Salsbury TI Underhill RM Flaherty JE Vlachokostas A. Investigation of potential aerosol transmission and infectivity of SARS-CoV-2 through central ventilation systems. Build Environ. (2021) 197:107633. doi: 10.1016/j.buildenv.2021.107633, PMID: 33531734 PMC7844370

[70] Azimi P Stephens B. HVAC filtration for controlling infectious airborne disease transmission in indoor environments: predicting risk reductions and operational costs. Build Environ. (2013) 70:150–60. doi: 10.1016/j.buildenv.2013.08.025, PMID: 32288024 PMC7127325

[71] Jankowska E Reponen T Willeke K Grinshpun SA Choi KJ. Collection of fungal spores on air filters and spore reentrainment from filters into air. J Aerosol Sci. (2000) 31:969–78. doi: 10.1016/S0021-8502(00)00017-3

[72] Greatorex JS Digard P Curran MD Moynihan R Wensley H Wreghitt T. Survival of influenza a(H1N1) on materials found in households: implications for infection control. PLoS One. (2011) 6:e27932. doi: 10.1371/journal.pone.0027932, PMID: 22132172 PMC3222642

[73] Kasloff SB Leung A Strong JE Funk D Cutts T. Stability of SARS-CoV-2 on critical personal protective equipment. Sci Rep. (2021) 11:80098. doi: 10.1038/s41598-020-80098-3, PMID: 33441775 PMC7806900

[74] Ji JH Bae GN Yun SH Jung JH Noh HS Kim SS. Evaluation of a silver nanoparticle generator using a small ceramic heater for inactivation of *S. epidermidis* bioaerosols. Aerosol Sci Technol. (2007) 41:786–93. doi: 10.1080/02786820701459932

[75] Hwang GB Heo KJ Yun JH Lee JE Lee HJ Nho CW. Antimicrobial air filters using natural Euscaphis japonica nanoparticles. PLoS One. (2015) 10:e0126481. doi: 10.1371/journal.pone.0126481, PMID: 25974109 PMC4431859

[76] Jung JH Hwang GB Lee JE Bae GN. Preparation of airborne ag/CNT hybrid nanoparticles using an aerosol process and their application to antimicrobial air filtration. Langmuir. (2011) 27:10256–64. doi: 10.1021/la201851r, PMID: 21751779

[77] Lee BU Yun SH Jung JH Bae GN. Effect of relative humidity and variation of particle number size distribution on the inactivation effectiveness of airborne silver nanoparticles against bacteria bioaerosols deposited on a filter. J Aerosol Sci. (2010) 41:447–56. doi: 10.1016/j.jaerosci.2010.02.005

[78] Huang R Pyankov OV Yu B Agranovski IE. Inactivation of fungal spores collected on fibrous filters by melaleuca alternifolia (tea tree oil). Aerosol Sci Technol. (2010) 44:262–8. doi: 10.1080/02786820903580188

[79] Sim KM Park HS Bae GN Jung JH. Antimicrobial nanoparticle-coated electrostatic air filter with high filtration efficiency and low pressure drop. Sci Total Environ. (2015) 533:266–74. doi: 10.1016/j.scitotenv.2015.07.003, PMID: 26172593

[80] Pyankov OV Usachev EV Pyankova O Agranovski IE. Inactivation of airborne influenza virus by tea tree and eucalyptus oils. Aerosol Sci Technol. (2012) 46:1295–302. doi: 10.1080/02786826.2012.708948

[81] Pyankov OV Agranovski IE Huang R Mullins BJ. Removal of biological aerosols by oil coated filters. Clean (Weinh). (2008) 36:609–14. doi: 10.1002/clen.200700191

[82] Peters TM Sawvel RA Park JH Anthony TR. Evaluation of a shaker dust collector for use in a recirculating ventilation system. J Occup Environ Hyg. (2015) 12:D201–10. doi: 10.1080/15459624.2015.1043056, PMID: 25955507 PMC4753559

[83] Dee S Otake S Deen J. Use of a production region model to assess the efficacy of various air filtration systems for preventing airborne transmission of porcine reproductive and respiratory syndrome virus and *Mycoplasma hyopneumoniae*: results from a 2-year study. Virus Res. (2010) 154, 22. doi: 10.1016/j.virusres.2010.07.022, PMID: 20667494

[84] Dee S Batista L Deen J Pijoan C. Evaluation of an air-filtration system for preventing aerosol transmission of porcine reproductive and respiratory syndrome virus. Can J Vet Res. (2005) 69:293–8. PMID: 16479728 PMC1250242

[85] Wenke C Pospiech J Reutter T Truyen U Speck S. Efficiency of different air filter types for pig facilities at laboratory scale. PLoS One. (2017) 12:6558. doi: 10.1371/journal.pone.0186558, PMID: 29028843 PMC5640248

[86] Wenke C Pospiech J Reutter T Altmann B Truyen U Speck S. Impact of different supply air and recirculating air filtration systems on stable climate, animal health, and performance of fattening pigs in a commercial pig farm. PLoS One. (2018) 13:e0194641. doi: 10.1371/journal.pone.0194641, PMID: 29558482 PMC5860761

[87] Alavy M Siegel JA. In-situ effectiveness of residential HVAC filters. Indoor Air. (2020) 30:156–66. doi: 10.1111/ina.12617, PMID: 31665545

[88] Adamiak K. Numerical models in simulating wire-plate electrostatic precipitators: a review. J Electrost. (2013) 71:673–80. doi: 10.1016/j.elstat.2013.03.001

[89] Mizuno A. Electrostatic precipitation. IEEE Trans Dielectr Electr Insul. (2000) 7:615–24. doi: 10.1109/94.879357, PMID: 37890623

[90] Qu J Zeng M Zhang D Yang D Wu X Ren Q. A review on recent advances and challenges of ionic wind produced by corona discharges with practical applications. J Phys D Appl Phys. (2022) 55:153002. doi: 10.1088/1361-6463/ac3e2c

[91] Chang JS Lawless PA Yamamoto T. Corona discharge processes. IEEE Trans Plasma Sci. (1991) 19:1152–66. doi: 10.1109/27.125038

[92] Zhuang Y Jin Kim Y Gyu Lee T Biswas P. Experimental and theoretical studies of ultra-fine particle behavior in electrostatic precipitators. J Electrost. (2000) 48:245–60. doi: 10.1016/S0304-3886(99)00072-8

[93] Morán J Li L Ouyang H Qiao Y Olson BA Hogan CJ. Characterization of the bidimensional size and charge distribution of sub- and supermicrometer particles in an electrostatic precipitator. Powder Technol. (2023) 425:118578. doi: 10.1016/j.powtec.2023.118578

[94] Gopalakrishnan R Thajudeen T Ouyang H Hogan CJ. The unipolar diffusion charging of arbitrary shaped aerosol particles. J Aerosol Sci. (2013) 64:60–80. doi: 10.1016/j.jaerosci.2013.06.002

[95] Liu BYH Kapadia A. Combined field and diffusion charging of aerosol particles in the continuum regime. J Aerosol Sci. (1978) 9:227–42. doi: 10.1016/0021-8502(78)90045-9

[96] Lawless PA Sparks LE. Modeling particulate charging in ESPs. IEEE Trans Ind Appl. (1988) 24:922–7. doi: 10.1109/28.8999, PMID: 36336044

[97] White HJ. Role of electrostatic precipitators in particulate control: a retrospective and prospective view. J Air Pollut Control Assoc. (1975) 25:102–7. doi: 10.1080/00022470.1975.10470052

[98] Jaworek A Marchewicz A Sobczyk AT Krupa A Czech T. Two-stage electrostatic precipitators for the reduction of PM2.5 particle emission. Progress Ener Combustion Sci. (2018) 67:206–33. doi: 10.1016/j.pecs.2018.03.003

[99] Jaworek A Sobczyk AT Krupa A Marchewicz A Czech T Śliwiński L. Hybrid electrostatic filtration systems for fly ash particles emission control. A review. Separat Purificat Technol. (2019) 213:283–302. doi: 10.1016/j.seppur.2018.12.011

[100] Afshari A Ekberg L Forejt L Mo J Rahimi S Siegel J. Electrostatic precipitators as an indoor air cleaner— A literature review. Sustainability. (2020) 12:1–20. doi: 10.3390/su1221877435136666

[101] Priyamvada H Kumaragama K Chrzan A Athukorala C Sur S Dhaniyala S. Design and evaluation of a new electrostatic precipitation-based portable low-cost sampler for bioaerosol monitoring. Aerosol Sci Technol. (2021) 55:24–36. doi: 10.1080/02786826.2020.1812503

[102] Lee SA Willeke K Mainelis G Adhikari A Wang H Reponen T. Assessment of electrical charge on airborne microorganisms by a new bioaerosol sampling method. J Occup Environ Hyg. (2004) 1:127–38. doi: 10.1080/15459620490424357, PMID: 15204870

[103] Miller A Frey G King G Sunderman C. A handheld electrostatic precipitator for sampling airborne particles and nanoparticles. Aerosol Sci Technol. (2010) 44:417–27. doi: 10.1080/02786821003692063

[104] Morrow PE Mercer TT. A point-to-plane electrostatic precipitator for particle size sampling. Am Ind Hyg Assoc J. (2007) 25:8–14. doi: 10.1080/00028896409342547 14110683

[105] Li CS Wen YM. Control effectiveness of electrostatic precipitation on airborne microorganisms. Aerosol Sci Technol. (2003) 37:933–8. doi: 10.1080/02786820300903, PMID: 25695127

[106] Kakutani K Matsuda Y Nonomura T Takikawa Y Takami T Toyoda H. A simple electrostatic precipitator for trapping virus particles spread via droplet transmission. Int J Environ Res Public Health. (2021) 18:4934. doi: 10.3390/ijerph18094934, PMID: 34066356 PMC8124561

[107] Kettleson EM Ramaswami B Hogan CJ Lee MH Statyukha GA Biswas P. Airborne virus capture and inactivation by an electrostatic particle collector. Environ Sci Technol. (2009) 43:5940–6. doi: 10.1021/es803289w, PMID: 19731701

[108] Mainelis G Górny RL Reponen T Trunov M Grinshpun SA Baron P. Effect of electrical charges and fields on injury and viability of airborne bacteria. Biotechnol Bioeng. (2002) 79:229–41. doi: 10.1002/bit.10290, PMID: 12115440

[109] Gao W Wang Y Zhang H Guo B Zheng C Guo J. Numerical simulation of particle migration in electrostatic precipitator with different electrode configurations. Powder Technol. (2020) 361:238–47. doi: 10.1016/j.powtec.2019.08.046

[110] Poppendieck DG Rim D Persily AK. Ultrafine particle removal and ozone generation by in-duct electrostatic precipitators. Environ Sci Technol. (2014) 48:2067–74. doi: 10.1021/es404884p, PMID: 24387032

[111] Chen TM Tsai CJ Yan SY Li SN. An efficient wet electrostatic precipitator for removing nanoparticles, submicron and micron-sized particles. Sep Purif Technol. (2014) 136:27–35. doi: 10.1016/j.seppur.2014.08.032

[112] Bologa A Paur HR Seifert H Wäscher T Woletz K. Novel wet electrostatic precipitator for collection of fine aerosol. J Electrost. (2009) 67:150–3. doi: 10.1016/j.elstat.2009.01.059

[113] Wu B Tian H Hao Y Liu S Liu X Liu W. Effects of wet flue gas desulfurization and wet electrostatic precipitators on emission characteristics of particulate matter and its ionic compositions from four 300 MW level ultralow coal-fired power plants. Environ Sci Technol. (2018) 52:14015–26. doi: 10.1021/acs.est.8b03656, PMID: 30378426

[114] Feng Z Long Z Yu T. Filtration characteristics of fibrous filter following an electrostatic precipitator. J Electrost. (2016) 83:52–62. doi: 10.1016/j.elstat.2016.07.009

[115] Lee M Koziel JA Macedo N Li P Chen B Jenks WS. Mitigation of particulate matter and airborne pathogens in swine barn emissions with filtration and UV-A photocatalysis. Catalysts. (2021) 11:1302. doi: 10.3390/catal11111302

[116] Center for Disease Control and Prevention. Upper-room ultraviolet germicidal irradiation (UVGI). National Center for Immunization and Respiratory Diseases (NCIRD), Division of Viral Diseases (2021).

[117] Brickner PW Vincent RL First M Nardell E Murray M Kaufman W. The application of ultraviolet germicidal irradiation to control transmission of airborne disease: bioterrorism countermeasure. Public Health Rep. (2003) 118:99–114. doi: 10.1016/S0033-3549(04)50225-X, PMID: 12690064 PMC1497517

[118] Koller LR. Ultraviolet radiation, vol. 2. New York: Wiley (1965).

[119] Tseng CC Li CS. Inactivation of virus-containing aerosols by ultraviolet germicidal irradiation. Aerosol Sci Technol. (2005) 39:1136–42. doi: 10.1080/02786820500428575, PMID: 33524450

[120] Azuma K Yanagi U Kagi N Kim H Ogata M Hayashi M. Environmental factors involved in SARS-CoV-2 transmission: Effect and role of indoor environmental quality in the strategy for COVID-19 infection control. Environ Health Prev Med. (2020) 25:66. doi: 10.1186/s12199-020-00904-2 33143660 PMC7607900

[121] Wang C Lu S Zhang Z. Inactivation of airborne bacteria using different UV sources: performance modeling, energy utilization, and endotoxin degradation. Sci Total Environ. (2019) 655:787–95. doi: 10.1016/j.scitotenv.2018.11.266, PMID: 30481706 PMC7112078

[122] Reed NG. The history of ultraviolet germicidal irradiation for air disinfection. Public Health Rep. (2010) 125:15–27. doi: 10.1177/003335491012500105, PMID: 20402193 PMC2789813

[123] Nardell E Vincent R Sliney DH. Upper-room ultraviolet germicidal irradiation (UVGI) for air disinfection: a symposium in print. Photochem Photobiol. (2013) 89:764–9. doi: 10.1111/php.12098, PMID: 23683092

[124] Jensen MM. Inactivation of airborne viruses by ultraviolet irradiation. Appl Microbiol. (1964) 12:418–20. doi: 10.1128/am.12.5.418-420.1964, PMID: 14215971 PMC1058147

[125] Jensen MM. Bacteriophage aerosol challenge of installed air contamination control systems. Appl Microbiol. (1967) 15:1447–9. doi: 10.1128/am.15.6.1447-1449.1967 16349762 PMC547227

[126] Qiao Y Yang M Marabella IA McGee DAJ Aboubakr H Goyal S. Greater than 3-log reduction in viable coronavirus aerosol concentration in ducted ultraviolet-C (UV-C) systems. Environ Sci Technol. (2021) 55:4174–82. doi: 10.1021/acs.est.0c05763, PMID: 33263988

[127] Li P Koziel JA Macedo N Zimmerman JJ Wrzesinski D Sobotka E. Evaluation of an air cleaning device equipped with filtration and UV: comparison of removal efficiency on particulate matter and viable airborne bacteria in the inlet and treated air. Int J Environ Res Public Health. (2022) 19:16135. doi: 10.3390/ijerph192316135, PMID: 36498208 PMC9735963

[128] Thornton GM Fleck BA Fleck N Kroeker E Dandnayak D Zhong L. The impact of heating, ventilation, and air conditioning design features on the transmission of viruses, including the 2019 novel coronavirus: A systematic review of ultraviolet radiation. PLoS ONE. (2022) 17, 17:e0266487. doi: 10.1371/journal.pone.0266487 35395010 PMC8992995

[129] Luo H Zhong L. Ultraviolet germicidal irradiation (UVGI) for in-duct airborne bioaerosol disinfection: review and analysis of design factors. Build Environ. (2021) 197:107852. doi: 10.1016/j.buildenv.2021.107852 33846664 PMC8021448

[130] Eadie E Hiwar W Fletcher L Tidswell E O’Mahoney P Buonanno M. Far-UVC (222 nm) efficiently inactivates an airborne pathogen in a room-sized chamber. Sci Rep. (2022) 12:4373. doi: 10.1038/s41598-022-08462-z, PMID: 35322064 PMC8943125

[131] Buonanno M Welch D Shuryak I Brenner DJ. Far-UVC light (222 nm) efficiently and safely inactivates airborne human coronaviruses. Sci Rep. (2020) 10:67211. doi: 10.1038/s41598-020-67211-2, PMID: 32581288 PMC7314750

[132] Ramos CCR Roque JLA Sarmiento DB Suarez LEG Sunio JTP Tabungar KIB. Use of ultraviolet-C in environmental sterilization in hospitals: a systematic review on efficacy and safety. Int J Health Sci. (2020) 14:52–65. PMID: 33192232 PMC7644456

[133] Ruston C Zhang J Scott J Zhang M Graham K Linhares D. Efficacy of ultraviolet C exposure for inactivating Senecavirus a on experimentally contaminated surfaces commonly found on swine farms. Vet Microbiol. (2021) 256:109040. doi: 10.1016/j.vetmic.2021.109040, PMID: 33812295

[134] Lee M Koziel JA Murphy W Jenks WS Chen B Li P. Mitigation of odor and gaseous emissions from swine barn with UV-A and UV-C photocatalysis. Atmosphere. (2021) 12:585. doi: 10.3390/atmos12050585

[135] Lee M Koziel JA Murphy W Jenks WS Fonken B Storjohann R. Design and testing of mobile laboratory for mitigation of gaseous emissions from livestock agriculture with photocatalysis. Int J Environ Res Public Health. (2021) 18:1–22. doi: 10.3390/ijerph18041523 PMC7915192 33562692

[136] Lee M Li P Koziel JA Ahn H Wi J Chen B. Pilot-scale testing of UV-A light treatment for mitigation of NH3, H2S, GHGs, VOCs, odor, and O3 inside the poultry barn. Front Chem. (2020) 8:8. doi: 10.3389/fchem.2020.00613 32903735 PMC7438853

[137] Eisenlöffel L Reutter T Horn M Schlegel S Truyen U Speck S. Impact of UVC-sustained recirculating air filtration on airborne bacteria and dust in a pig facility. PLoS One. (2019) 14:e0225047. doi: 10.1371/journal.pone.0225047, PMID: 31697778 PMC6837447

[138] Li P Koziel JA Zimmerman JJ Zhang J Cheng TY Yim-Im W. Mitigation of airborne PRRSV transmission with UV light treatment: Proof-of-concept. Agriculture. (2021) 11:259. doi: 10.3390/agriculture11030259

[139] Houston JM Zarobila CJ Yoon HW Xu G Huang X. (2000). Characterization and calibration of broadband ultraviolet radiometers you may also like achievement of 0.005% combined transfer uncertainties in the NIST detector calibration facility characterization and calibration of broadband ultraviolet radiometers (Metrologia). 10.1088/1681-7575/ac499e PMC9793687 36578474

[140] Liu F Ma Q Marjub MM Suthammanont AK Sun S Yao H. Reactive air disinfection technologies: principles and applications in bioaerosol removal. ACS ES&T. Engineering. (2023) 3:602–15. doi: 10.1021/acsestengg.3c00016

[141] Zhang Y Mo J Li Y Sundell J Wargocki P Zhang J. Can commonly-used fan-driven air cleaning technologies improve indoor air quality? A literature review. Atmos Environ. (2011) 45:4329–43. doi: 10.1016/j.atmosenv.2011.05.041 PMC7185562 32362761

[142] Lee SG Hyun J Hwa Lee S Hwang J. One-pass antibacterial efficacy of bipolar air ions against aerosolized *Staphylococcus epidermidis* in a duct flow. J Aerosol Sci. (2014) 69:71–81. doi: 10.1016/j.jaerosci.2013.12.005

[143] Agranovski I Huang R Pyankov O Altman I Grinshpun S. Enhancement of the performance of low-efficiency HVAC filters due to continuous unipolar ion emission. Aerosol Sci Technol. (2006) 40:963–8. doi: 10.1080/02786820600833203, PMID: 18333990

[144] Huang R Agranovski I Pyankov O Grinshpun S. Removal of viable bioaerosol particles with a low-efficiency HVAC filter enhanced by continuous emission of unipolar air ions. Indoor Air. (2008) 18:106–12. doi: 10.1111/j.1600-0668.2007.00512.x, PMID: 18333990

[145] Grinshpun SA Mainelis G Trunov M Adhikari A Reponen T Willeke K. Evaluation of ionic air purifiers for reducing aerosol exposure in confined indoor spaces. Indoor Air. (2005) 15:235–45. doi: 10.1111/j.1600-0668.2005.00364.x, PMID: 15982270

[146] Pushpawela B Jayaratne R Nguy A Morawska L. Efficiency of ionizers in removing airborne particles in indoor environments. J Electrost. (2017) 90:79–84. doi: 10.1016/j.elstat.2017.10.002, PMID: 36474607

[147] Kolarž P Ilić AŽ Janković M Janićijević A Trbovich AM. Estimating aerosol particle removal in indoor air by ion-enhanced deposition. J Aerosol Sci. (2023) 173:106199. doi: 10.1016/j.jaerosci.2023.106199

[148] Hagbom M Nordgren J Nybom R Hedlund KO Wigzell H Svensson L. Ionizing air affects influenza virus infectivity and prevents airborne-transmission. Sci Rep. (2015) 5:5. doi: 10.1038/srep11431 PMC4477231 26101102

[149] Noh KC Lee JH Kim C Yi S Hwang J Yoon YH. Filtration of submicron aerosol particles using a carbon fiber ionizer-assisted electret filter. Aerosol Air Qual Res. (2011) 11:811–21. doi: 10.4209/aaqr.2011.05.0060

[150] Hyun J Lee SG Hwang J. Application of corona discharge-generated air ions for filtration of aerosolized virus and inactivation of filtered virus. J Aerosol Sci. (2017) 107:31–40. doi: 10.1016/j.jaerosci.2017.02.004, PMID: 32226115 PMC7094352

[151] Kim YS Yoon KY Park JH Hwang J. Application of air ions for bacterial de-colonization in air filters contaminated by aerosolized bacteria. Sci Total Environ. (2011) 409:748–55. doi: 10.1016/j.scitotenv.2010.11.012, PMID: 21146197

[152] Park JH Yoon KY Kim YS Byeon JH Hwang J. Removal of submicron aerosol particles and bioaerosols using carbon fiber ionizer assisted fibrous medium filter media. J Mech Sci Technol. (2009) 23:1846–51. doi: 10.1007/s12206-009-0613-z

[153] Li J Gao H Lan C Nie L Liu D Lu X. Plasma air filtration system for intercepting and inactivation of pathogenic microbial aerosols. J Environ Chem Eng. (2023) 11:110728. doi: 10.1016/j.jece.2023.110728, PMID: 37200549

[154] Nouri H Zouzou N Moreau E Dascalescu L Zebboudj Y. Effect of relative humidity on current-voltage characteristics of an electrostatic precipitator. J Electrost. (2012) 70:20–4. doi: 10.1016/j.elstat.2011.08.011

[155] Li M Ma Z Xia J Zhang C He X Zhao L. Investigation of humid air on Corona onset voltage in wire-plane electrodes under AC-DC composite voltages via test and modeling. IEEE Trans Dielectr Electr Insul. (2023) 30:1105–14. doi: 10.1109/TDEI.2023.3248528

[156] Xu P Zhang B Chen S He J. Influence of humidity on the characteristics of positive corona discharge in air. Phys Plasmas. (2016) 23:890. doi: 10.1063/1.4953890, PMID: 22715745

[157] Zeng Y Manwatkar P Laguerre A Beke M Kang I Ali AS. Evaluating a commercially available in-duct bipolar ionization device for pollutant removal and potential byproduct formation. Build Environ. (2021) 195:107750. doi: 10.1016/j.buildenv.2021.107750

[158] Nunayon SS Zhang HH Jin X Lai AC. Experimental evaluation of positive and negative air ions disinfection efficacy under different ventilation duct conditions. Build Environ. (2019) 158:295–301. doi: 10.1016/j.buildenv.2019.05.027

[159] Park JH Yoon KY Hwang J. Removal of submicron particles using a carbon fiber ionizer-assisted medium air filter in a heating, ventilation, and air-conditioning (HVAC) system. Build Environ. (2011) 46:1699–708. doi: 10.1016/j.buildenv.2011.02.010

[160] Kang Y Kato S. Thermal and non-thermal germicidal effects of microwave radiation on microbial agents. Indoor Built Environ. (2014) 23:1080–91. doi: 10.1177/1420326X13490180

[161] Wu Y Yao M. Inactivation of bacteria and fungus aerosols using microwave irradiation. J Aerosol Sci. (2010) 41:682–93. doi: 10.1016/j.jaerosci.2010.04.004

[162] Shaw P Kumar N Mumtaz S Lim JS Jang JH Kim D. Evaluation of non-thermal effect of microwave radiation and its mode of action in bacterial cell inactivation. Sci Rep. (2021) 11:14003. doi: 10.1038/s41598-021-93274-w, PMID: 34234197 PMC8263747

[163] Wang C Hu X Zhang Z. Airborne disinfection using microwave-based technology: energy efficient and distinct inactivation mechanism compared with waterborne disinfection. J Aerosol Sci. (2019) 137:105437. doi: 10.1016/j.jaerosci.2019.105437 32226120 PMC7094417

[164] Wu Y Yao M. In situ airborne virus inactivation by microwave irradiation. Chin Sci Bull. (2014) 59:1438–45. doi: 10.1007/s11434-014-0171-3, PMID: 32214745 PMC7089037

[165] Betti L Trebbi G Lazzarato L Brizzi M Calzoni GL Marinelli F. Nonthermal microwave radiations affect the hypersensitive response of tobacco to tobacco mosaic virus. J Altern Complement Med. (2004) 10:947–57. doi: 10.1089/acm.2004.10.947, PMID: 15673988

[166] Wu Y Yao M. Control of airborne and liquid-borne fungal and pet allergens using microwave irradiation. J Occup Environ Hyg. (2013) 10:547–55. doi: 10.1080/15459624.2013.818234, PMID: 24011331

[167] Woo MH Grippin A Wu CY Wander J. Microwave-irradiation-assisted HVAC filtration for inactivation of viral aerosols. Aerosol Air Qual Res. (2012) 12:295–303. doi: 10.4209/aaqr.2011.11.0193

[168] Lin CY Li CS. Effectiveness of titanium dioxide photocatalyst filters for controlling bioaerosols. Aerosol Sci Technol. (2003) 37:162–70. doi: 10.1080/02786820300951

[169] Poormohammadi A Bashirian S Rahmani AR Azarian G Mehri F. Are photocatalytic processes effective for removal of airborne viruses from indoor air? A narrative review. Environ Sci Pollut Res. (2021) 28:43007–20. doi: 10.1007/s11356-021-14836-z, PMID: 34128162 PMC8203310

[170] Habibi-Yangjeh A Asadzadeh-Khaneghah S Feizpoor S Rouhi A. Review on heterogeneous photocatalytic disinfection of waterborne, airborne, and foodborne viruses: can we win against pathogenic viruses? J Colloid Interface Sci Acad. (2020) 580:503–14. doi: 10.1016/j.jcis.2020.07.047, PMID: 32711201 PMC7361121

[171] Zhang C Li Y Shuai D Shen Y Wang D. Progress and challenges in photocatalytic disinfection of waterborne viruses: a review to fill current knowledge gaps. Chem Eng J. (2019) 355:399–415. doi: 10.1016/j.cej.2018.08.158

[172] Nakata K Fujishima A. TiO 2 photocatalysis: design and applications. J Photochem Photobiol C: Photochem Rev. (2012) 13:169–89. doi: 10.1016/j.jphotochemrev.2012.06.001

[173] Gunschera J Markewitz D Bansen B Salthammer T Ding H. Portable photocatalytic air cleaners: efficiencies and by-product generation. Environ Sci Pollut Res. (2016) 23:7482–93. doi: 10.1007/s11356-015-5992-3, PMID: 26711293

[174] Dumont É Héquet V. Determination of the clean air delivery rate (CADR) of photocatalytic oxidation (PCO) purifiers for indoor air pollutants using a closed-loop reactor. Part I: Theoretical Considerations Molecules. Molecules. (2017) 22:407. doi: 10.3390/molecules22030407 28272308 PMC6155194

[175] Zacarías SM Satuf ML Vaccari MC Alfano OM. Efficiency evaluation of different TiO_2_ coatings on the photocatalytic inactivation of airborne bacterial spores. Ind Eng Chem Res. (2012) 51:13599–608. doi: 10.1021/ie3009956

[176] Qiao Y Yang M Marabella IA McGee DAJ Olson BA Torremorell M. Wind tunnel-based testing of a photoelectrochemical oxidative filter-based air purification unit in coronavirus and influenza aerosol removal and inactivation. Indoor Air. (2021) 31:2058–69. doi: 10.1111/ina.12847, PMID: 33960547 PMC8242653

[177] Chuaybamroong P Chotigawin R Supothina S Sribenjalux P Larpkiattaworn S Wu CY. Efficacy of photocatalytic HEPA filter on microorganism removal. Indoor Air. (2010) 20:246–54. doi: 10.1111/j.1600-0668.2010.00651.x, PMID: 20573124

[178] Denny F Permana E Scott J Wang J Pui DYH Amal R. Integrated photocatalytic filtration array for indoor air quality control. Environ Sci Technol. (2010) 44:5558–63. doi: 10.1021/es100421u, PMID: 20550189

[179] Ginestet A Pugnet D Rowley J Bull K Yeomans H. Development of a new photocatalytic oxidation air filter for aircraft cabin. Indoor Air. (2005) 15:326–34. doi: 10.1111/j.1600-0668.2005.00369.x, PMID: 16108905

[180] Bono N Ponti F Punta C Candiani G. Effect of UV irradiation and TiO2-photocatalysis on airborne bacteria and viruses: An overview. Materials. (2021) 14:1–20. doi: 10.3390/ma14051075 PMC7956276 33669103

[181] Hay SO Obee T Luo Z Jiang T Meng Y He J. The viability of photocatalysis for air purification. Molecules. (2015) 20:1319–56. doi: 10.3390/molecules20011319 25594345 PMC6272289

[182] Schneider J Matsuoka M Takeuchi M Zhang J Horiuchi Y Anpo M. Understanding TiO2photocatalysis: mechanisms and materials. Chem Rev. (2014) 114:9919–86. doi: 10.1021/cr5001892 25234429

[183] Kang SU Choi JW Chang JW Kim K Kim YS Park JK. N_2_ non-thermal atmospheric pressure plasma promotes wound healing *in vitro* and *in vivo*: potential modulation of adhesion molecules and matrix metalloproteinase-9. Exp Dermatol. (2017) 26:163–70. doi: 10.1111/exd.13229, PMID: 27673439

[184] Ishaq M Evans MM Ostrikov KK. Effect of atmospheric gas plasmas on cancer cell signaling. Int J Cancer. (2014) 134:1517–28. doi: 10.1002/ijc.28323, PMID: 23754175

[185] Fridman G Friedman G Gutsol A Shekhter AB Vasilets VN Fridman A. Applied plasma medicine. Plasma Process Polym. (2008) 5:503–33. doi: 10.1002/ppap.200700154, PMID: 37901550

[186] Wang G Zhu R Yang L Wang K Zhang Q Su X. Non-thermal plasma for inactivated-vaccine preparation. Vaccine. (2016) 34:1126–32. doi: 10.1016/j.vaccine.2015.10.099, PMID: 26529075

[187] Šimončicová J Kryštofová S Medvecká V Ďurišová K Kaliňáková B. Technical applications of plasma treatments: Current state and perspectives. Appl Microbiol Biotechnol. (2019) 103:5117–29. doi: 10.1007/s00253-019-09877-x 31089766

[188] Assadi I Guesmi A Baaloudj O Zeghioud H Elfalleh W Benhammadi N. Review on inactivation of airborne viruses using non-thermal plasma technologies: from MS2 to coronavirus. Environ Sci Pollut Res Int. (2022) 29:4880–92. doi: 10.1007/s11356-021-17486-3 34796437 PMC8601095

[189] Chen Z Garcia G Arumugaswami V Wirz RE. Cold atmospheric plasma for SARS-CoV-2 inactivation. Phys Fluids. (2020) 32:1332. doi: 10.1063/5.0031332, PMID: 33244211 PMC7684674

[190] Filipić A Gutierrez-Aguirre I Primc G Mozetič M Dobnik D. Cold plasma, a new Hope in the field of virus inactivation. Trends Biotechnol. (2020) 38:1278–91. doi: 10.1016/j.tibtech.2020.04.003 32418663 PMC7164895

[191] Mohamed H Nayak G Rendine N Wigdahl B Krebs FC Bruggeman PJ. Non-thermal plasma as a novel strategy for treating or preventing viral infection and associated disease. Front Phys. (2021) 9. doi: 10.3389/fphy.2021.683118

[192] Kaushik N Mitra S Baek EJ Nguyen LN Bhartiya P Kim JH. The inactivation and destruction of viruses by reactive oxygen species generated through physical and cold atmospheric plasma techniques: current status and perspectives. J Adv Res. (2022) 43:59–71. doi: 10.1016/j.jare.2022.03.002 36585115 PMC8905887

[193] Schiappacasse C Peng P Zhou N Liu X Zhai J Cheng Y. Inactivation of aerosolized Newcastle disease virus with non-thermal plasma. Appl Eng Agric. (2020) 36:55–60. doi: 10.13031/aea.13699

[194] Wu Y Liang Y Wei K Li W Yao M Zhang J. MS2 virus inactivation by atmospheric-pressure cold plasma using different gas carriers and power levels. Appl Environ Microbiol. (2015) 81:996–1002. doi: 10.1128/AEM.03322-14, PMID: 25416775 PMC4292470

[195] Xia T Kleinheksel A Lee EM Qiao Z Wigginton KR Clack HL. Inactivation of airborne viruses using a packed bed non-thermal plasma reactor. J Phys D Appl Phys. (2019) 52:1466. doi: 10.1088/1361-6463/ab1466, PMID: 32287389 PMC7106774

[196] Xia T Yang M Marabella I Lee EM Olson B Zarling D. Inactivation of airborne porcine reproductive and respiratory syndrome virus (PRRSv) by a packed bed dielectric barrier discharge non-thermal plasma. J Hazard Mater. (2020) 393:122266. doi: 10.1016/j.jhazmat.2020.122266, PMID: 32126420

[197] Nayak G Andrews AJ Marabella I Aboubakr HA Goyal SM Olson BA. Rapid inactivation of airborne porcine reproductive and respiratory syndrome virus using an atmospheric pressure air plasma. Plasma Process Polym. (2020) 17:269. doi: 10.1002/ppap.201900269

[198] Nayak G Oinuma G Yue Y Sousa JS Bruggeman PJ. Plasma-droplet interaction study to assess transport limitations and the role of ·OH, O·,H·,O2(a1Δg),O3, He(23S) and Ar(1s5) in formate decomposition. Plasma Sources Sci Technol. (2021) 30:2676. doi: 10.1088/1361-6595/ac2676

[199] Nayak G Du Y Brandenburg R Bruggeman PJ. Effect of air flow on the micro-discharge dynamics in an array of integrated coaxial microhollow dielectric barrier discharges. Plasma Sources Sci Technol. (2017) 26:035001. doi: 10.1088/1361-6595/aa56a4

[200] Moldgy A Nayak G Aboubakr HA Goyal SM Bruggeman PJ. Inactivation of virus and bacteria using cold atmospheric pressure air plasmas and the role of reactive nitrogen species. J Phys D Appl Phys. (2020) 53:434004. doi: 10.1088/1361-6463/aba066

[201] Zhang L Guo Y Chang X Yao Z Wei X Feng Z. In-duct grating-like dielectric barrier discharge system for air disinfection. J Hazard Mater. (2022) 435:129075. doi: 10.1016/j.jhazmat.2022.129075, PMID: 35650753 PMC9072810

[202] Sutton D Aldous EW Warren CJ Fuller CM Alexander DJ Brown IH. Inactivation of the infectivity of two highly pathogenic avian influenza viruses and a virulent Newcastle disease virus by ultraviolet radiation. Avian Pathol. (2013) 42:566–8. doi: 10.1080/03079457.2013.853867, PMID: 24188498

[203] Huber TW Brackens E Chatterjee P Villamaria FC Sisco LE Williams MD. Efficacy of pulsed-xenon ultraviolet light on reduction of *Mycobacterium fortuitum*. SAGE Open Med. (2020) 8:205031212096237. doi: 10.1177/2050312120962372 PMC7550950 33101679

[204] Noyce JO Hughes JF. Bactericidal effects of negative and positive ions generated in nitrogen on *Escherichia coli*. J Electrost. (2002) 54:179–87. doi: 10.1016/S0304-3886(01)00179-6, PMID: 32080228

[205] Dos Santos T de Castro LF. Evaluation of a portable ultraviolet C (UV-C) device for hospital surface decontamination. Photodiagn Photodyn Ther. (2021) 33:102161. doi: 10.1016/j.pdpdt.2020.102161, PMID: 33373741 PMC7764389

[206] Hogan CJ Kettleson EM Lee MH Ramaswami B Angenent LT Biswas P. Sampling methodologies and dosage assessment techniques for submicrometre and ultrafine virus aerosol particles. J Appl Microbiol. (2005) 99:1422–34. doi: 10.1111/j.1365-2672.2005.02720.x, PMID: 16313415

[207] Ahmadi Y Bhardwaj N Kim KH Kumar S. Recent advances in photocatalytic removal of airborne pathogens in air. Sci Total Environ. (2021) 794:148477. doi: 10.1016/j.scitotenv.2021.148477 34198079

[208] Barnewall RE Bischoff WE. Removal of SARS-CoV-2 bioaerosols using ultraviolet air filtration. Infect Control Hosp Epidemiol. (2021) 42:1014–5. doi: 10.1017/ice.2021.103, PMID: 33706834 PMC8007938

[209] Prehn F Timmermann E Kettlitz M Schaufler K Günther S Hahn V. Inactivation of airborne bacteria by plasma treatment and ionic wind for indoor air cleaning. Plasma Process Polym. (2020) 17:27. doi: 10.1002/ppap.202000027

[210] Farnsworth JE Goyal SM Won Kim S Kuehn TH Raynor PC Ramakrishnan MA. Development of a method for bacteria and virus recovery from heating, ventilation, and air conditioning (HVAC) filters. J Environ Monit. (2006) 8:1006. doi: 10.1039/b606132j, PMID: 17240906

[211] Nirmala J Alves G Vilalta C Yang M Rendahl A Olson B. Evaluation of viral RNA extraction methods to detect porcine reproductive and respiratory syndrome and influenza a viruses from used commercial HVAC air filters from swine farms. J Aerosol Sci. (2021) 151:105624. doi: 10.1016/j.jaerosci.2020.105624

[212] Noris F Siegel JA Kinney KA. Evaluation of HVAC filters as a sampling mechanism for indoor microbial communities. Atmos Environ. (2011) 45:338–46. doi: 10.1016/j.atmosenv.2010.10.017

[213] Ouyang H Qiao Y Yang M Marabella IA Hogan CJ Torremorell M. Single-pass wind tunnel testing for recirculating virus aerosol control technologies. J Aerosol Sci. (2022) 165:106045. doi: 10.1016/j.jaerosci.2022.106045

[214] AHAM. Method for measuring performance of portable household electric room air cleaners. Association of Home Appliance Manufacturers. (2015).

[215] Liu CY Tseng CH Wang HC Dai CF Shih YH. The study of an ultraviolet radiation technique for removal of the indoor air volatile organic compounds and bioaerosol. Int J Environ Res Public Health. (2019) 16. doi: 10.3390/ijerph16142557, PMID: 31319616 PMC6678761

[216] Staszowska A. Assessment of the air purifier effectiveness under model conditions. J. Phys. Conf. Ser. (2021) 1736:012043. doi: 10.1088/1742-6596/1736/1/012043

[217] Lee JH Kim JY Cho BB Anusha JR Sim JY Raj CJ. Assessment of air purifier on efficient removal of airborne bacteria, *Staphylococcus epidermidis*, using single-chamber method. Environ Monit Assess. (2019) 191:7876. doi: 10.1007/s10661-019-7876-3, PMID: 31691038 PMC7087645

[218] Mainka A Mucha W Pastuszka JS Bragoszewska E Janoszek A. Non-commercial air purifier-the effectiveness and safety. Buildings. (2020) 10:104. doi: 10.3390/buildings10060104, PMID: 28755565

[219] Dbouk T Roger F Drikakis D. Reducing indoor virus transmission using air purifiers. Phys Fluids. (2021) 33:103301. doi: 10.1063/5.0064115, PMID: 34629834 PMC8498854

[220] Dee SA Deen J Cano JP Batista L Pijoan C. Further evaluation of alternative air-filtration systems for reducing the transmission of porcine reproductive and respiratory syndrome virus by aerosol. Can J Vet Res. (2006) 70:168–75. PMID: 16850938 PMC1477932

[221] Dee SA Batista L Deen J Pijoan C. Evaluation of systems for reducing the transmission of porcine reproductive and respiratory syndrome virus by aerosol. Can J Vet Res. (2006) 70:28–33. 16548329 PMC1325091

[222] Torremorell M Pijoan C Janni K Walker R Joo HS. Airborne transmission of Actinobacillus pleuropneumoniae and porcine reproductive and respiratory syndrome virus in nursery pigs. Am J Vet Res. (1997) 58:828–32. 9256964

[223] Dee S Torremorell M Thompson B Deen J Pijoan C. An evaluation of thermo-assisted drying and decontamination for the elimination of porcine reproductive and respiratory syndrome virus from contaminated livestock transport vehicles. Can J Vet Res. (2005) 69:58–63. 15745224 PMC1142171

[224] Alonso C Davies PR Polson DD Dee SA Lazarus WF. Financial implications of installing air filtration systems to prevent PRRSV infection in large sow herds. Prev Vet Med. (2013) 111:268–77. doi: 10.1016/j.prevetmed.2013.05.001 23735427

[225] Nagaraj S Chandrasingh S Jose S Sofia B Sampath S Krishna B. Effectiveness of a novel, non-intrusive, continuous-use air decontamination technology to reduce microbial contamination in clinical settings: a multi-centric study. J Hosp Infect. (2022) 123:15–22. doi: 10.1016/j.jhin.2022.02.002, PMID: 35181400

[226] Ereth MH Fine J Stamatatos F Mathew B Hess D Simpser E. Healthcare-associated infection impact with bioaerosol treatment and COVID-19 mitigation measures. J Hosp Infect. (2021) 116:69–77. doi: 10.1016/j.jhin.2021.07.006 34302883 PMC8295046

[227] Wu HT Li QS Dai RC Liu S Wu L Mao W. Effects of air-conditioning systems in the public areas of hospitals: a scoping review. Epidemiol Infect. (2021) 149:e201. doi: 10.1017/S0950268821001990

[228] May KR. The collison nebulizer: description, performance and application. J Aerosol Sci. (1973) 4:235–43.

[229] Danelli SG Brunoldi M Massabò D Parodi F Vernocchi V Prati P. Comparative characterization of the performance of bio-aerosol nebulizers in connection with atmospheric simulation chambers. Atmos Meas Tech. (2021) 14:4461–70. doi: 10.5194/amt-14-4461-2021

[230] Hermann JR Hoff SJ Yoon KJ Burkhardt AC Evans RB Zimmerman JJ. Optimization of a sampling system for recovery and detection of airborne porcine reproductive and respiratory syndrome virus and swine influenza virus. Appl Environ Microbiol. (2006) 72:4811–8. doi: 10.1128/AEM.00472-06, PMID: 16820475 PMC1489351

[231] Gautam US Asrican R Sempowski GD. Targeted dose delivery of *Mycobacterium tuberculosis* in mice using silicon antifoaming agent via aerosol exposure system. PLoS One. (2022) 17:e0276130. doi: 10.1371/journal.pone.0276130 36228009 PMC9560519

[232] Tessum MW Raynor PC. Measuring electrostatic charge on pneumatically generated spray drops. J Aerosol Sci. (2021) 151:105691. doi: 10.1016/j.jaerosci.2020.105691

[233] Johnson GR Morawska L Ristovski ZD Hargreaves M Mengersen K Chao CYH. Modality of human expired aerosol size distributions. J Aerosol Sci. (2011) 42:839–51. doi: 10.1016/j.jaerosci.2011.07.009

[234] Mainelis G. Bioaerosol sampling: Classical approaches, advances, and perspectives. Aerosol Sci Technol. (2020) 54:496–519. doi: 10.1080/02786826.2019.1671950 35923417 PMC9344602

[235] Lindsley WG Green BJ Blachere FM Martin SB Law BF Jensen PA. Sampling and characterization of bioaerosols [internet]. 5th Edn. NIOSH manual of analytical methods (NMAM). (2017). Available at: https://www.researchgate.net/publication/315706252 (Accessed November 2, 2023).

[236] Ghosh B Lal H Srivastava A. Review of bioaerosols in indoor environment with special reference to sampling, analysis and control mechanisms. Environ Int. (2015) 85:254–72. doi: 10.1016/j.envint.2015.09.018 26436919 PMC7132379

[237] Anderson BD Lednicky JA Torremorell M Gray GC. The use of bioaerosol sampling for airborne virus surveillance in swine production facilities: A mini review. Front Vet Sci. (2017) 4:121. doi: 10.3389/fvets.2017.00121 28798919 PMC5529434

[238] Manibusan S Mainelis G. Passive bioaerosol samplers: a complementary tool for bioaerosol research. A review. J Aerosol Sci Elsevier Ltd. (2022) 163:105992. doi: 10.1016/j.jaerosci.2022.105992 PMC9648171 36386279

[239] Cox J Mbareche H Lindsley WG Duchaine C. Field sampling of indoor bioaerosols. Aerosol Sci Technol. (2020) 54:572–84. doi: 10.1080/02786826.2019.1688759 31777412 PMC6880939

[240] Mbareche H Brisebois E Veillette M Duchaine C. Bioaerosol sampling and detection methods based on molecular approaches: no pain no gain. Sci Total Environ. (2017) 599–600:2095–104. doi: 10.1016/j.scitotenv.2017.05.076 28558432

[241] Pan M Lednicky JA Wu CY. Collection, particle sizing and detection of airborne viruses. J Appl Microbiol. (2019) 127:1596–611. doi: 10.1111/jam.14278 30974505 PMC7167052

[242] Chen P Du Y Xu YZ Liu Z Yan K. Review: bioaerosol collection. Int J Plasma Environ Sci Technol. (2017) 11:52–5.

[243] Verreault D Moineau S Duchaine C. Methods for sampling of airborne viruses. Microbiol Mol Biol Rev. (2008) 72:413–44. doi: 10.1128/MMBR.00002-08, PMID: 18772283 PMC2546863

[244] Rahmani AR Leili M Azarian G Poormohammadi A. Sampling and detection of corona viruses in air: a mini review. Sci Total Environ. (2020) 740:140207. doi: 10.1016/j.scitotenv.2020.140207 32554029 PMC7295527

[245] Mbareche H Veillette M Bilodeau GJ Duchaine C. Bioaerosol sampler choice should consider efficiency and ability of samplers to cover microbial diversity. Appl Environ Microbiol. (2018) 84:1589. doi: 10.1128/AEM.01589-18 PMC6238049 30217848

[246] Ratnesar-Shumate S Bohannon K Williams G Holland B Krause M Green B. Comparison of the performance of aerosol sampling devices for measuring infectious SARS-CoV-2 aerosols. Aerosol Sci Technol. (2021) 55:975–86. doi: 10.1080/02786826.2021.1910137 PMC10698689 38076006

[247] Su WC Tolchinsky AD Chen BT Sigaev VI Cheng YS. Evaluation of physical sampling efficiency for cyclone-based personal bioaerosol samplers in moving air environments. J Environ Monit. (2012) 14:2430–7. doi: 10.1039/c2em30299c, PMID: 22833144 PMC4649907

[248] Alonso C Raynor PC Davies PR Torremorell M. Concentration, size distribution, and infectivity of airborne particles carrying swine viruses. PLoS One. (2015) 10:e0135675. doi: 10.1371/journal.pone.0135675 26287616 PMC4545937

[249] King MD Lacey RE Pak H Fearing A Ramos G Baig T. Assays and enumeration of bioaerosols-traditional approaches to modern practices. Aerosol Sci Technol. (2020) 54:611–33. doi: 10.1080/02786826.2020.1723789

[250] Decaro N Elia G Campolo M Desario C Mari V Radogna A. Detection of bovine coronavirus using a TaqMan-based real-time RT-PCR assay. J Virol Methods. (2008) 151:167–71. doi: 10.1016/j.jviromet.2008.05.016, PMID: 18579223 PMC7112840

[251] Wathes CM Howard K Webster AJF. The survival of *Escherichia coli* in an aerosol at air temperatures of 15 and 30 IC and a range of humidities. J Hyg Camb. (1986) 97:489–96. doi: 10.1017/s0022172400063671 3540114 PMC2082889

[252] Spronk G Otake S Dee S. Prevention of PRRSV infection in large breeding herds using air filtration. Vet Rec. (2010) 166:758–9. doi: 10.1136/vr.b4848, PMID: 20543168

[253] Fisk WJ Faulkner D Palonen J Seppanen O. Performance and costs of particle air filtration technologies. Indoor Air. (2002) 12:223–34. doi: 10.1034/j.1600-0668.2002.01136.x, PMID: 12532754

[254] Stephens B. Wells-Riley & HVAC filtration for infectious airborne aerosols NAFA foundation report HVAC filtration and the Wells-Riley approach to assessing risks of infectious airborne diseases final report prepared for: The National air Filtration Association (NAFA) foundation 291 Independence [internet]. (2013). Available at: www.built-envi.com

[255] Fisk WJ Chan WR. Effectiveness and cost of reducing particle-related mortality with particle filtration. Indoor Air. (2017) 27:909–20. doi: 10.1111/ina.12371, PMID: 28170103

[256] Montgomery JF Green SI Rogak SN Bartlett K. Predicting the energy use and operation cost of HVAC air filters. Energ Buildings. (2012) 47:643–50. doi: 10.1016/j.enbuild.2012.01.001, PMID: 25952610

[257] Bekö G Clausen G Weschler CJ. Is the use of particle air filtration justified? Costs and benefits of filtration with regard to health effects, building cleaning and occupant productivity. Build Environ. (2008) 43:1647–57. doi: 10.1016/j.buildenv.2007.10.006

[258] Montgomery JF Reynolds CCO Rogak SN Green SI. Financial implications of modifications to building filtration systems. Build Environ. (2015) 85:17–28. doi: 10.1016/j.buildenv.2014.11.005

[259] Riley EC Murphy G Riley RL. Airborne spread of measles in a suburban elementary school. Am J Epidemiol. (1978) 107:421–32. doi: 10.1093/oxfordjournals.aje.a112560, PMID: 665658

[260] Knibbs LD Morawska L Bell SC Grzybowski P. Room ventilation and the risk of airborne infection transmission in 3 health care settings within a large teaching hospital. Am J Infect Control. (2011) 39:866–72. 21658810 10.1016/j.ajic.2011.02.014 PMC7115323

[261] Carmen Alonso Garcia-Mochales B. Concentration, size distribution, and control of swine viruses associated with airborne particles. St Paul: University of Minnesota (2016). doi: 10.1016/j.ajic.2011.02.014

